# Vascular changes in hypertrophic cardiomyopathy

**DOI:** 10.1093/cvr/cvag153

**Published:** 2026-07-15

**Authors:** Julia E Visch, Ali Nassar, George Burchell, Tjeerd Germans, Jolanda van der Velden

**Affiliations:** Department of Physiology, Amsterdam Cardiovascular Sciences, Amsterdam UMC, Location VUmc, O2 Science building, De Boelelaan 1108, 1081 HZ, Amsterdam, The Netherlands; Department of Cardiology, Amsterdam Cardiovascular Sciences, Amsterdam UMC, Location VUmc, De Boelelaan 1117, 1081 HV, Amsterdam, The Netherlands; Department of Physiology, Amsterdam Cardiovascular Sciences, Amsterdam UMC, Location VUmc, O2 Science building, De Boelelaan 1108, 1081 HZ, Amsterdam, The Netherlands; Medical Library, Vrije Universiteit Amsterdam, De Boelelaan 1105, 1081 HV Amsterdam, The Netherlands; Department of Physiology, Amsterdam Cardiovascular Sciences, Amsterdam UMC, Location VUmc, O2 Science building, De Boelelaan 1108, 1081 HZ, Amsterdam, The Netherlands; Department of Cardiology, Amsterdam Cardiovascular Sciences, Amsterdam UMC, Location VUmc, De Boelelaan 1117, 1081 HV, Amsterdam, The Netherlands; Department of Cardiology, Noordwest Ziekenhuisgroep, Wilhelminalaan 12, 1815 JD, Alkmaar, The Netherlands; Department of Physiology, Amsterdam Cardiovascular Sciences, Amsterdam UMC, Location VUmc, O2 Science building, De Boelelaan 1108, 1081 HZ, Amsterdam, The Netherlands; Department of Experimental Cardiology, Amsterdam Cardiovascular Sciences, Amsterdam UMC, Location AMC, Meibergdreef 9, 1105 AZ, Amsterdam, The Netherlands; Netherlands Heart Institute, Moreelsepark 1, 3511 EP, Utrecht, The Netherlands

**Keywords:** Hypertrophic cardiomyopathy, Subclinical HCM, Microvascular changes, Endothelial dysfunction, Myocardial perfusion

## Abstract

**Aims:**

Hypertrophic cardiomyopathy (HCM) is characterized by left ventricular hypertrophy along with (micro)vascular abnormalities. These include cardiac and peripheral vascular dysfunction, as well as structural vascular changes. This review summarizes the modalities for assessing (micro)vascular function and explores how vascular changes relate to disease progression, including structural remodelling, fibrosis, and clinical outcomes.

**Methods and results:**

A systematic search of multiple databases was conducted to identify studies investigating vascular changes in patients with HCM, varying in disease severity. Human studies were included without time restrictions and studies involving hypertrophy secondary to other conditions (e.g. hypertension, aortic stenosis) were excluded.

A total of 130 studies were included. Vascular changes were assessed with Positron Emission Tomography (*n* = 24), Cardiac Magnetic Resonance Imaging (*n* = 21), angiography (*n* = 18), scintigraphy and Single Photon Emission Computed Tomography (*n* = 30), peripheral measures (*n* = 12), and histology (*n* = 29). Perfusion abnormalities were common in HCM. While resting myocardial blood flow appears to be similar between HCM patients and controls, myocardial perfusion reserve is consistently reduced, correlating with early disease features, structural remodelling, and adverse outcomes. Peripheral vascular dysfunction is also frequently observed, though findings vary and mechanisms remain unclear.

**Conclusion:**

Vascular abnormalities play a key role in the pathophysiology of HCM and are closely linked to disease progression and prognosis. Data are limited regarding the role of vascular dysfunction in genotype-positive/phenotype-negative individuals and individuals with early or mild disease. Future research should focus on evaluating cardiac and peripheral vascular function across the disease spectrum to better understand underlying mechanisms and potential targets for intervention.

## Graphical Abstract

**Graphical Abstract:**
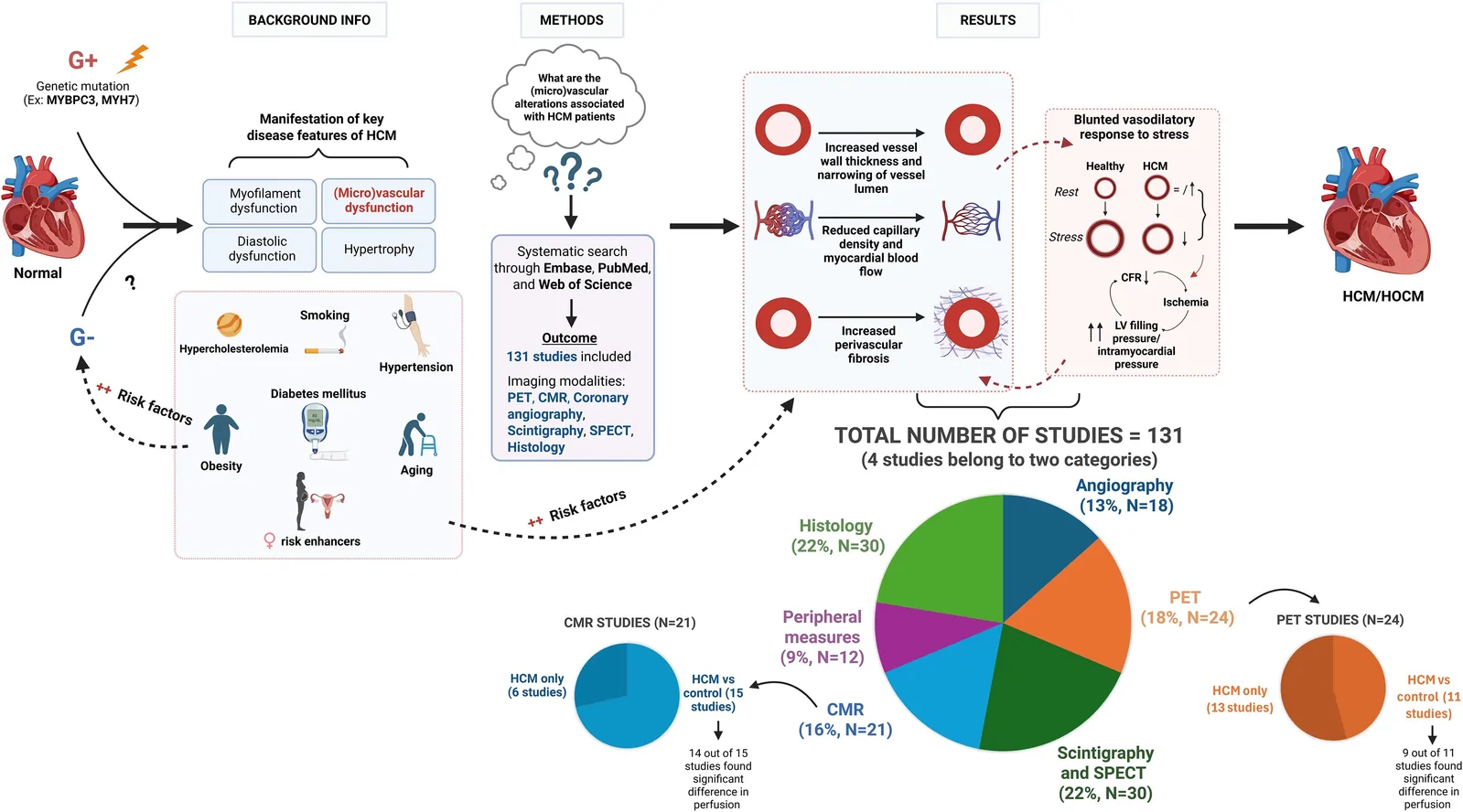

**Time of primary review: 74 days**

## 1. Introduction

Hypertrophic cardiomyopathy (HCM) is an autosomal dominant genetic disease, characterized by (asymmetrical) hypertrophy of the left ventricle (LV) not solely explained by the presence of increased loading conditions such as systemic hypertension of aortic valve stenosis. The most common pathogenic and likely pathogenic (P/LP) gene variants involved in HCM lie in the sarcomere genes *MYBPC3*, *MYH7*, and *TNNT2*.^1^ Clinical manifestations observed in HCM patients are diastolic dysfunction, hypercontractility, LV outflow tract (LVOT) obstruction, chest pain, arrhythmias, and heart failure. Penetrance and disease severity vary substantially. To date, little is known about the factors that contribute to the variable disease course.

Histopathological characteristics of HCM include myocyte disarray, fibrosis, and microvascular alterations.^2^ Changes in microvasculature include reduced capillary density, medial hypertrophy, intimal hyperplasia, a decreased luminal size, and stenosis of the microcirculation.^3–5^ Moreover, changes in microvascular function occur even before the onset of hypertrophy,^6^ suggesting that microvascular dysfunction may contribute to variable disease severity and progression. A recent study shows evidence for cross-talk between mutant-harbouring cardiomyocytes and endothelial cells.^7^

**Figure 1:**
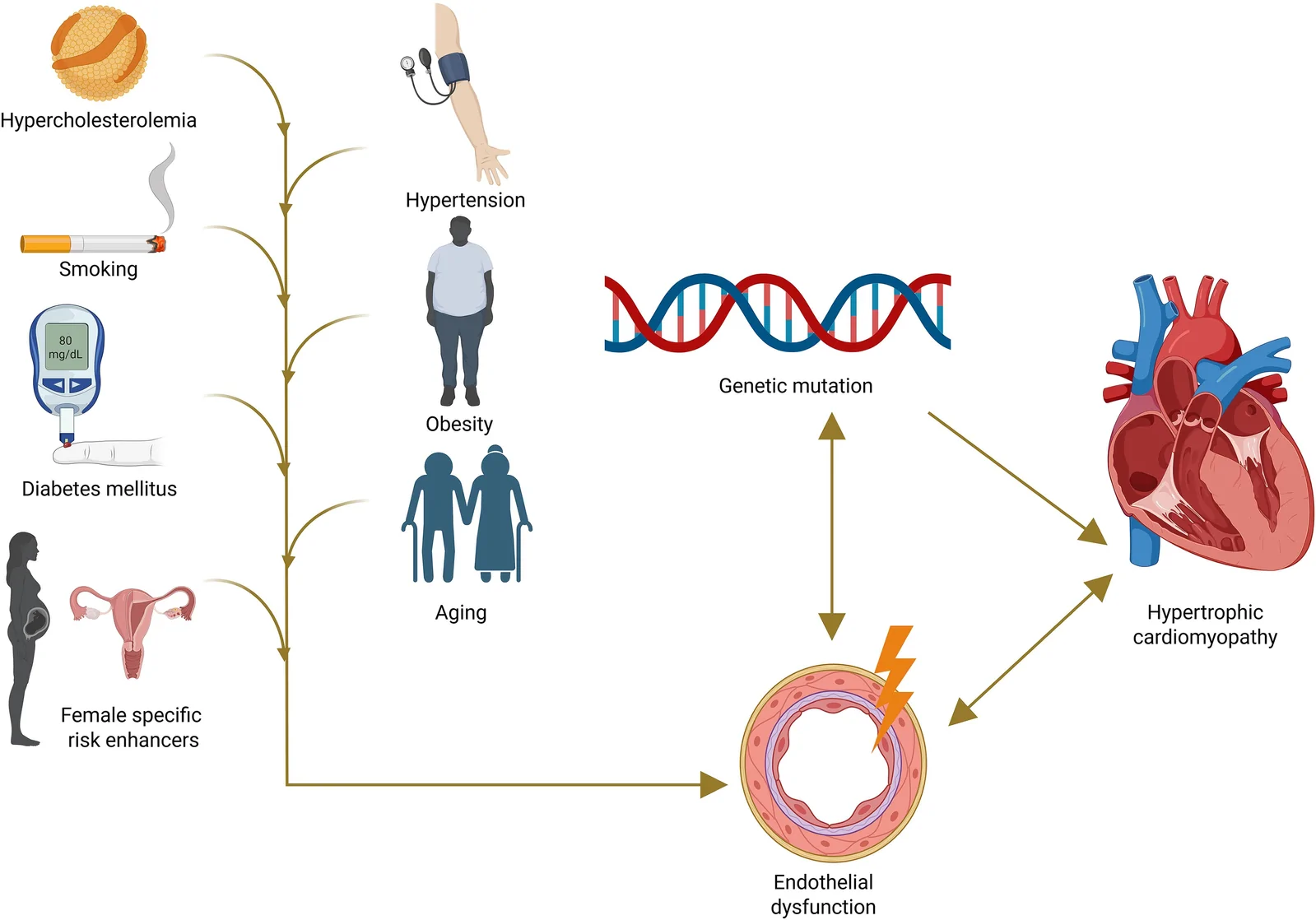
Complex interplay among genotype, different risk factors, and vascular changes in the initiation and progression of hypertrophic cardiomyopathy.

Interestingly, not only the cardiac but also peripheral endothelial cell function was shown to be impaired in HCM patients.^8^ Endothelial function is influenced by factors such as obesity, female-specific risk enhancers, hypercholesterolaemia, smoking, diabetes mellitus, ageing, and hypertension.^9–11^ The complex interplay between these different risk factors, with a central role for vascular endothelial dysfunction in HCM development is poorly understood (*Figure 1*). This systematic review aims to (i) provide an overview of the different modalities and methods that are used to assess vascular (and endothelial) changes, and (ii) gives an overview of cardiac and peripheral vascular changes that have been reported in HCM, which may underlie initiation and progression of disease.

## 2. Methods

### 2.1 Search strategy

A systematic search was performed on 15 April 2023 through multiple databases (PubMed, Embase, Web of Science). Supplementary material online, *Table S1* provides a detailed description of search criteria. Our review research question was ‘Which vascular changes are present in patients during the course of hypertrophic cardiomyopathy?’ Thereto, we included studies reporting on patients with mild to overt hypertrophy and genotype-positive/phenotype-negative (G+/Ph−) individuals. We defined HCM according to the AHA guidelines; maximal end-diastolic wall thickness of ≥15 mm anywhere in the LV, without another cause of hypertrophy in adults. Less hypertrophy (13–14 mm) was diagnosed as HCM when found in family members of a patient with HCM, or in patients with a positive genetic test.^12^ For this review, we included both G+ and genotype-negative (G−) patients, and all forms of HCM (obstructive HCM, apical and septal hypertrophy). Reported vascular changes included both cardiac and peripheral alterations. The review was based on human studies and we did not limit the inclusion to a certain time period.

The exclusion criteria were: hypertrophy caused by aortic stenosis or hypertension, patient population with a mean age of <18 years, papers assessing obstructive coronary artery disease, a study population of less than five patients, full text not in English, case reports, reviews, retracted articles, and letters to the editor. Papers that mentioned vascular changes in HCM, but did not provide a separate report or analysis of the HCM patient group, thus reporting on a mixed patient group including other aetiologies, were excluded. Studies assessing myocardial perfusion and/or coronary flow were only included when positron emission Tomography (PET), Cardiac Magnetic Resonance (CMR), coronary angiography, scintigraphy, or Single-photon emission computed tomography (SPECT) was used. Only papers available in full text were included. To ensure a homogenous patient population, we excluded studies where hypertension (systolic blood pressure >140 mmHg or diastolic blood pressure >90 mmHg) was not excluded or if hypertension was reported in the baseline characteristics. Abstracts that were included in the initial search and, at the time of writing this review, had a full-text publication were included.

## 3. Results

### 3.1 Search results

**Figure 2:**
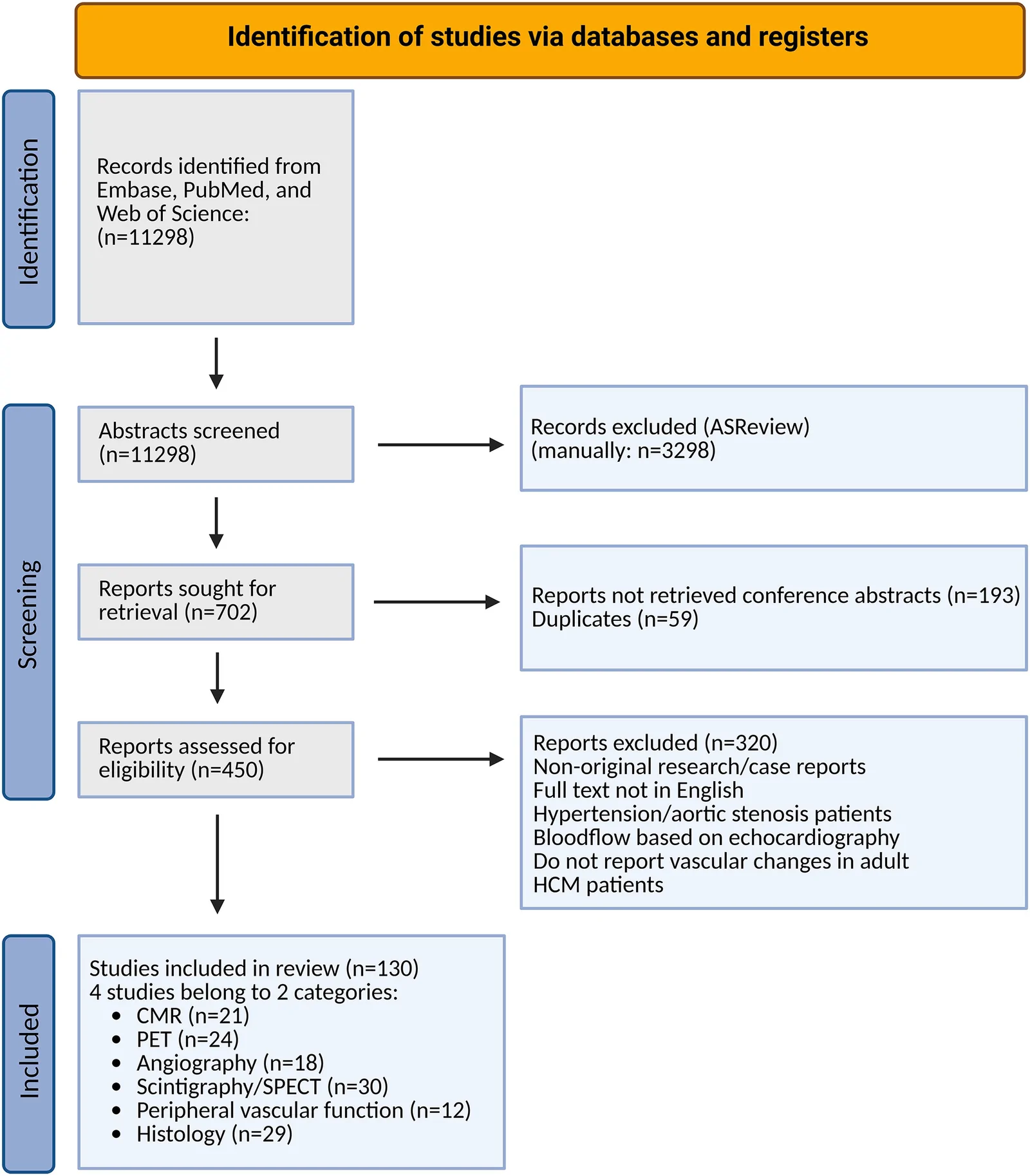
PRISMA flow chart. HCM, hypertrophic cardiomyopathy; CMR, cardiac magnetic resonance; PET, positron emission tomography; SPECT, single-photon emission computed tomography.

**Figure 3:**
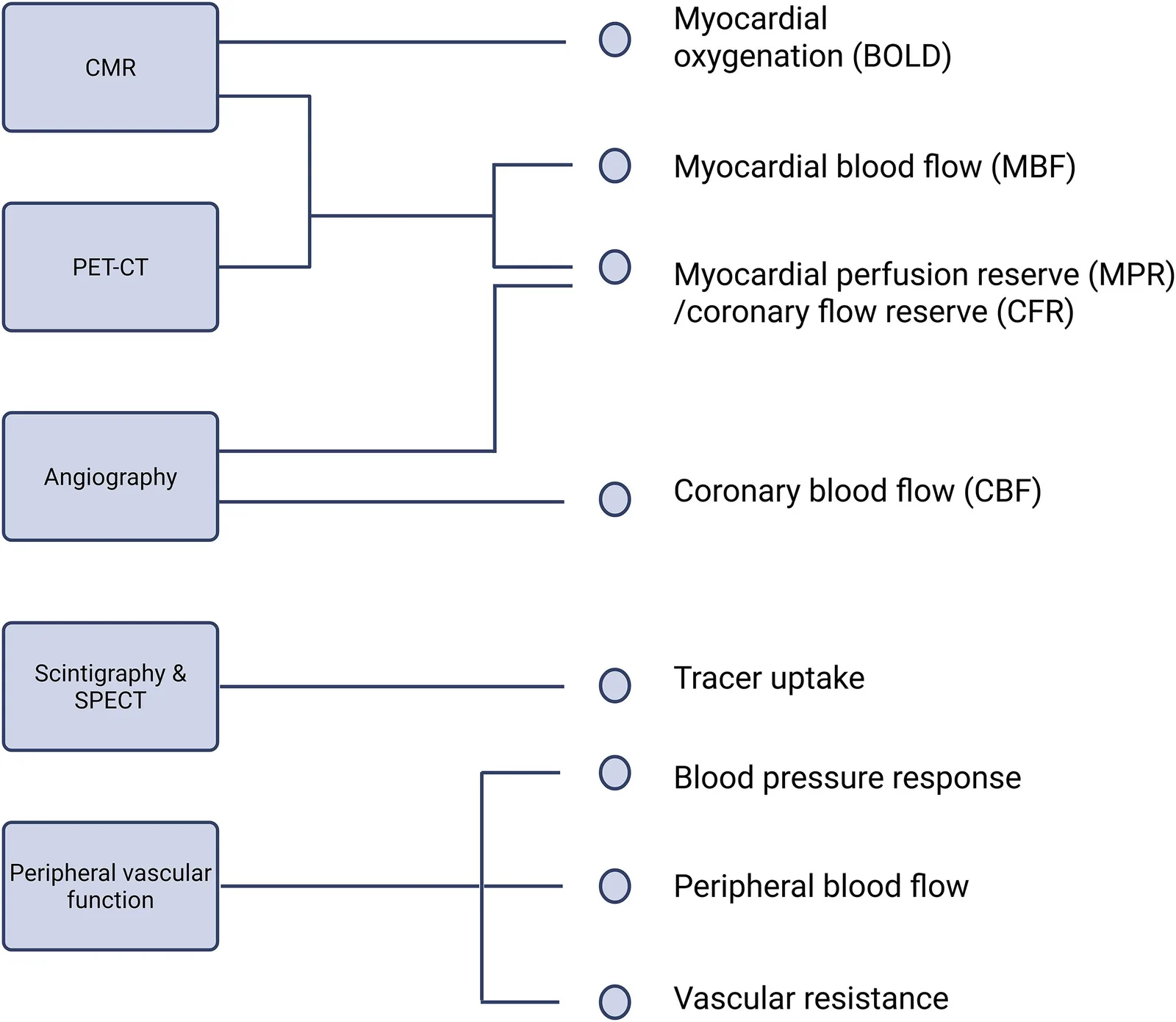
Overview of included modalities and their parameters.

**Figure 4:**
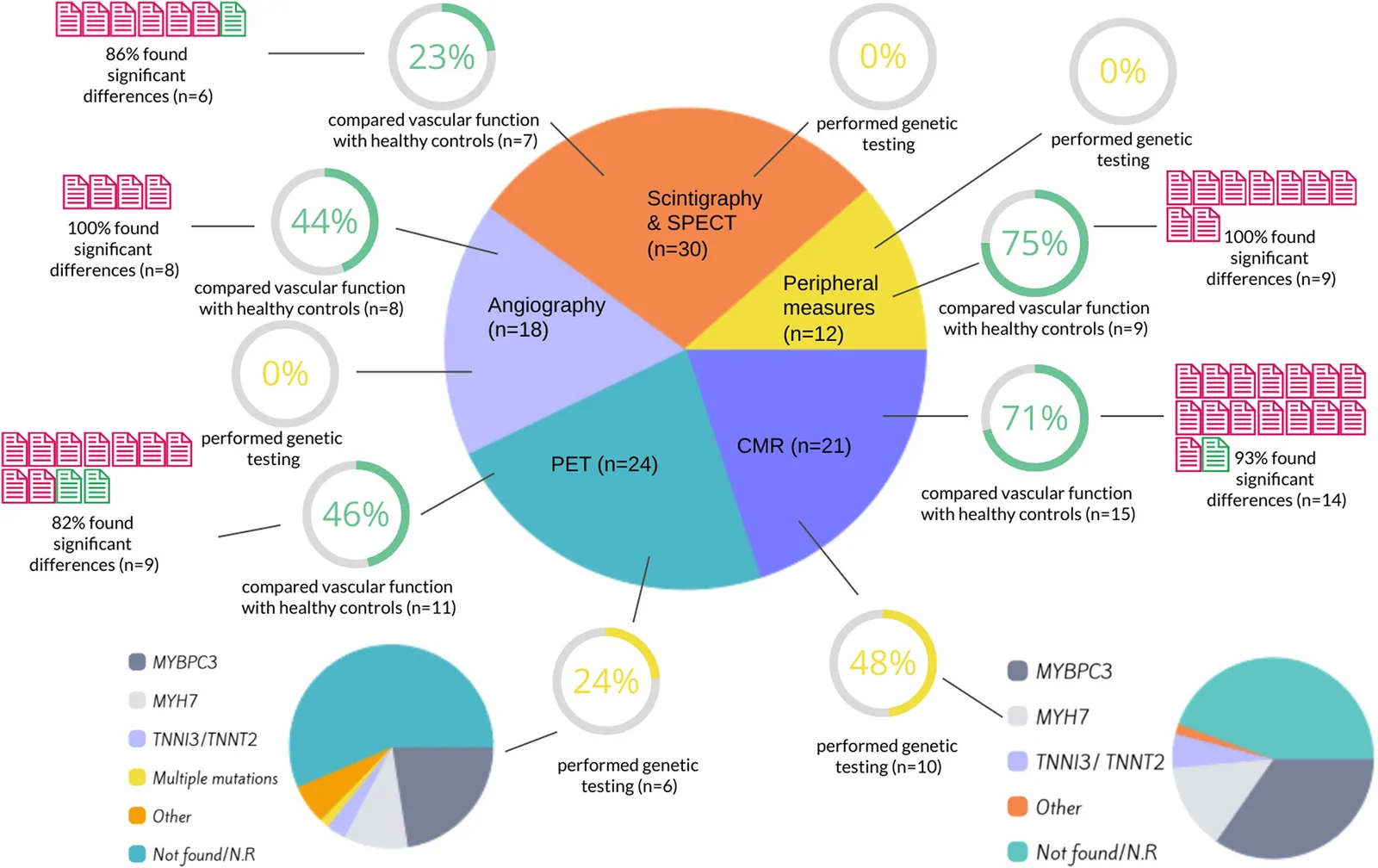
Overview of the included studies. CMR, cardiac magnetic resonance; PET, positron emission tomography; SPECT, single-photon emission computed tomography; MYBPC3, myosin binding protein C3; MYH7, myosin heavy chain 7; TNNI3, troponin I3; TTNT2, troponin T2.

The database search identified 11.298 articles. After screening the abstracts using ASReview^13^ 702 full texts were assessed, of which 130 papers were included.^3–5,14–141^ A flow diagram is illustrated in *Figure 2*. An overview of the included modalities and their parameters is shown in *Figure 3*. *Figure 4* shows the main findings of each modality. A formal risk of bias assessment was performed for each study (see Supplementary material online, *Tables S4* and *S5*).

### 3.2 Vascular changes in HCM patients

#### 3.2.1 Positron emission tomography

**Figure 5:**
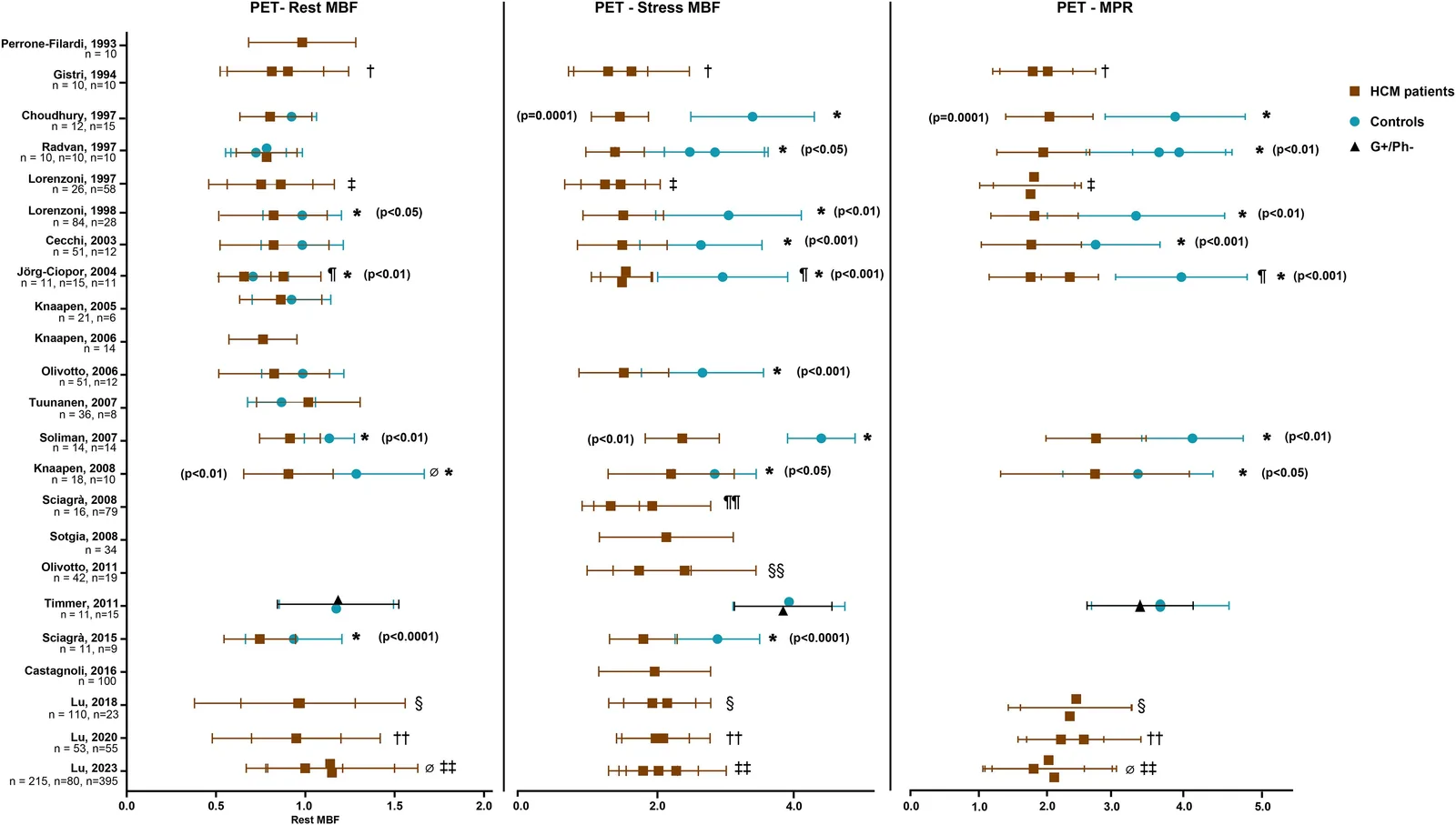
Overview of PET studies reporting on (*A*) rest MBF, (*B*) stress MBF, and (*C*) CFR. All values are mean ± SD. Statistical analysis using the Student’s *t*-test was performed in Choudhury *et al.*, Cecchi *et al.*, Olivotto *et al.*, Soliman *et al.*, Knaapen *et al.*, and Sciagrà *et al.*; and using ANOVA in Radvan *et al.*, Lorenzoni *et al.*, Cecchi *et al.*, and Jörg-Ciopor *et al.* * = *P* < 0.05 for HCM vs. a control group. ⌀ = MBF corrected for LVRPP or RPP. † = HCM patients before treatment with placebo vs. HCM patients before treatment with Verapamil. ‡ = HCM patients with NSVT vs. HCM patients without NSVT. ¶ = HCM patients after myectomy vs. controls vs. medically treated HCM patients. § = HCM without ventricular arrythmias vs. HCM patients with ventricular arrythmias. †† = HCM patients without LV cavity dilation vs. HCM patients with LV cavity dilation. ‡‡ = HCM patients with a blood pressure > 140 mmHg vs. ≤110 mmHg vs. 110–140 mmHg. ¶¶ = HCM patients with atrial fibrillation vs. HCM patients without atrial fibrillation. §§ = Genotype positive HCM patients vs. genotype negative HCM patients. *n* refers to number of HCM and controls. See Supplementary material online, *Table S2* for group specifics.

Positron emission tomography is considered the gold standard for quantification of myocardial blood flow (MBF).^142^ Multiple tracers are used for MBF assessment including H_2_15O and 13N-labelled ammonia, Rubidium, and Flurpiridaz.^143^ We included 24 PET studies (see Supplementary material online, *Table S2*: study and patient characteristics). Eleven studies compared MBF in HCM patients with healthy controls, of which nine found significant differences (*Figure 4*). Of the 10 studies that assessed resting MBF, only 3 found significantly lower MBF values in HCM compared to controls, while reported hyperaemic blood flow (hMBF) (*n* = 9) or coronary flow reserve (CFR) (*n* = 7) values were all significantly lower in HCM compared to controls (*Figure 5*). The reduction in hMBF and CFR observed in HCM surpasses that seen in other causes of hypertrophy—such as aortic valve stenosis, hypertension, and athlete’s heart,^37,48^ and is not only limited to hypertrophied segments but also in non-hypertrophied regions.^51,57^

Genetic testing was performed in 6 out of 24 studies, resulting in 180 G+/Ph+ patients, mostly with *MYBPC3* (93) and *MYH7* variants (42). One study reported significantly lower hMBF in G+ than in G− patients, and over twice as many G− than G+ patients were in the highest tertile of hMBF.^42^ The same study assessed differences between pathogenic variants in the two thick filament genes *MYBPC3* and *MYH7*, and found no significant differences in average hMBF, though patients with pathogenic *MYBPC3* variants showed a broad range of flow values, ranging from 0.6 to 3.1 mL/min/g, while all patients with *MYH7* variants had a (diminished) flow below 2.0 mL/min/g.^42^

#### 3.2.2 Cardiovascular magnetic resonance imaging

**Figure 6:**
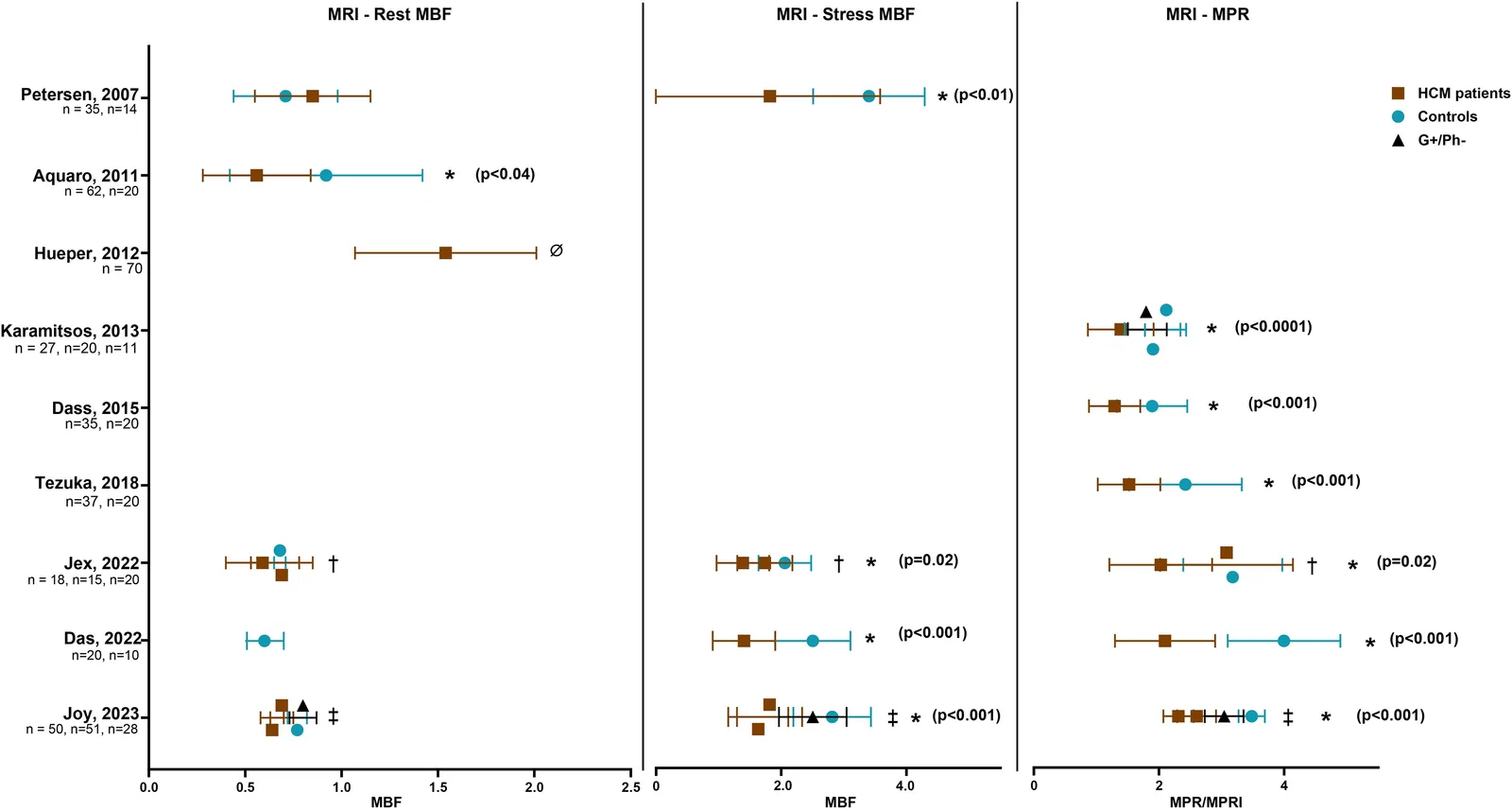
Overview of CMR studies reporting on (*A*) rest MBF, (*B*) stress MBF, and (*C*) CFR. All values are mean ± SD. Statistical analysis using the Student’s *t*-test was performed in Petersen *et al.*, Dass *et al.*, Tezuka *et al.*, Das *et al.*, and Joy *et al.*; and using ANOVA in Aquaro *et al.*, Karamitsos *et al.*, and Jex *et al.* * = *P* < 0.05 for HCM vs. a control group. ⌀ = MBF corrected for LVRPP or RPP. † = HCM patients without diabetes mellitus vs. controls vs. HCM patients with diabetes mellitus. ‡ = Genotype-negative HCM patients vs. genotype positive HCM patients vs. controls. *n* refers to number of HCM and controls. See Supplementary material online, *Table S3* for group specifics.

CMR allows the assessment of cardiac function and myocardial perfusion through multiple techniques.^144^ Besides myocardial perfusion, CMR can be employed to quantify fibrosis by late gadolinium enhancement (LGE), tissue characterization by mapping techniques, and visualization and quantification of flow patterns in the heart. We included 21 CMR studies.^14–34^  Supplementary material online, *Table S3* shows an overview of reported patient characteristics, cardiac dimensions, and measures of myocardial perfusion. First-pass perfusion was the most commonly used CMR technique to measure MBF during rest and stress, and was used to calculate myocardial perfusion reserve (MPR) or CFR (*Figure 3*). A total of 15 studies compared perfusion of HCM patients with controls,^14–18,20,21,23,24,27,29,31–34^ of which 14 studies reported significant perfusion differences between HCM and controls (*Figure 4*), either during rest or stress. Rest MBF in HCM patients was significantly lower in 1 out of the 4 studies reporting on rest MBF^18^ (*Figure 6*). Stress MBF and MPR were both significantly lower in all studies reporting on these parameters (four and six, respectively)^15,20,29,31–34^ (*Figure 6*), implying a blunted vasodilatory response. The reduced perfusion measures seem to be independent of age, as a decreased perfusion reserve index was already observed in young HCM patients (mean age 20 years).^17^

Genetic testing was performed in 10 out of 21 studies resulting in 242 G+/Ph+ individuals. Most pathogenic gene variants were in *MYBPC3* (*n* = 147), *MYH7* (*n* = 59), and *TNNI3* (*n* = 12). Two of the 10 studies did not report the outcome of the genetic testing.^15,20^ Sixty genetically tested HCM patients were G−/Ph+. Only one study compared G− with G+ HCM patients, and in this study, no difference in perfusion measures was found.^34^ No study assessed differences between different pathogenic variants or affected genes.

Another parameter reflecting coronary microvascular function is myocardial oxygenation, using the blood oxygen level dependent (BOLD) technique,^145,146^ and was applied in 2 out of the 21 studies.^20,21^ HCM patients exhibited an impaired oxygenation response during vasodilatory stress,^21^ which was accompanied by an impaired perfusion.^20^ A blunting of myocardial oxygenation was associated with impaired myocardial perfusion, increased wall thickness, and fibrosis.^20^

#### 3.2.3 Coronary angiography

Coronary blood flow (CBF) can be assessed invasively with coronary angiography at baseline and during maximal vasodilation/hyperaemia. This can be achieved using substances as acetylcholine (endothelium-dependent vasodilator), papaverine (mediated via smooth muscle cells), bradykinin (endothelium-dependent vasodilator), substance P (endothelium-dependent vasodilator), dobutamine (positive inotropic effect), and through rapid atrial pacing. Eighteen angiography studies were included. In none of the studies was genetic testing performed. Eight studies assessed differences in CBF between HCM patients and controls.^59–62,64,72,73,75^ Of these, 7 studies assessed CBF at rest: 4 found a higher rest CBF in HCM, 1 found a lower CBF in HCM, and 2 found comparable CBF values. Moreover, a decreased coronary resistance at rest was reported in 2 studies.^73,75^ Three studies assessed stress CBF, of which 2 reported a lower value in HCM compared to controls, and one did not find a difference. Of the five studies assessing CFR, all found a significantly lower value in HCM patients, suggesting a diminished vasodilatory capacity. Another finding supporting this theory is that during rapid-atrial pacing, HCM patients reach their maximal CBF earlier than healthy controls.^64,73^ Moreover, when the rate of atrial-pacing is increased further, the CBF eventually decreases.^73^ In addition, HCM patients have altered phasic coronary blood flow, flow reversal in systole, and an increased diastolic velocity time integral (a faster deceleration of diastolic CBF).^62–64,71,75^ This could also be due to increased intramyocardial pressure as it relates to the percentage change in diameter of the septal branches from diastole to systole, and HCM patients exhibit closure or narrowing of the septal vessels at end-systole.^62^ Overall, these results indicate a diminished vasodilatory capacity in HCM patients, with comparable resting CBF values and a diminished reserve.

One study showed that endothelium-dependent vasodilation was significantly lower in HCM patients,^60^ although this was not confirmed in another study.^61^ CBF or coronary diameter after the infusion of papaverine, nitroglycerin, or bradykinin did not differ from controls.^60,61^ In another study, no differences in CFR were found for papaverine, substance P and rapid atrial pacing, but the values were compared with controls from other studies, which hampers the interpretation of results.^63^ One study did find a decreased papaverine-induced CFR in HCM (with similar baseline CBF values).^61^

When comparing HCM patients with hypertensive LVH patients, the CBF and CFR values did not differ, but the diastolic/systolic velocity ratio (DSVR) was significantly higher in HCM patients. This was irrespective of LVM, again suggesting a different pathophysiological pathway of LVH, but could also be due to a lower systolic blood pressure and therefore reduced systolic blood flow (assuming a similar vascular resistance).^60^

Myocardial bridging (MB) and coronary spastic angina were more commonly found in HCM patients than in the average population or in patients with secondary hypertrophy.^67,68,70,71^ Ischaemia was not necessarily caused by myocardial bridging, as HCM patients without myocardial bridging still showed ischaemia.^76,80^ However, the incidence of septal perfusion defects increased as the grade of systolic compression of the major septal perforator increased, and exhibited a higher systolic retrograde flow velocity.^71^ HCM patients with abnormal thallium uptake showed significantly more frequent systolic compression of the septal perforator.^69^

#### 3.2.4 Scintigraphy and single photon emission computer tomography (SPECT)

Scintigraphy and SPECT use gamma-emitting tracers to assess myocardial perfusion. Examples of these tracers are Thallium-201, Tc-99m Sestamibi, and Tc-99m Tetrofosmin.^147^

Twelve scintigraphy studies and 18 SPECT studies were included. Genetic testing was performed in none of the studies. Perfusion deficits detected by SPECT and scintigraphy were common in HCM patients, as in the included studies 22–82% of HCM patients showed perfusion defects, either at rest or during stress/exercise,^80–85,87,92,96,98–100,102,141^ compared to absence of perfusion defects in controls.^85,141^ The reported percentage of patients with an irreversible perfusion defect varied between studies and amounted to 25 and 82%. Reversible perfusion defects occur in approximately 22–26%.^85,89,92^ SPECT imaging in apical HCM patients showed an increased tracer uptake at rest in the apical regions indicative of an increased apical blood flow.^93^ Hypertrophic obstructive cardiomyopathy (HOCM) patients also exhibited an increased tracer uptake, predominantly observed in the hypertrophied septum.^97^

Only three scintigraphy studies and four SPECT studies assessed differences in perfusion between HCM and controls, of which six found significantly more perfusion abnormalities in HCM patients.^69,79,85,94,101,141^ One study only found a decreased septal tracer washout rate in HCM patients, indicative of a reduced blood flow (BF), but no perfusion defects were seen.^101^ Another study did not find any difference in tracer uptake.^91^ Perfusion defects were most common in the inferoseptal and anteroseptal regions.^80,100^ The increase in Thallium-201 uptake after administration of dipyridamole was significantly blunted in HCM compared to controls and patients with hypertension.^69^ The blunted response was found in the septal segments as well as in the lateral segments. Notably, the septal segments exhibited a lower perfusion in HCM compared to hypertensive patients, even though the amount of hypertrophy was comparable.

#### 3.2.5 Link between perfusion abnormalities and structural and functional properties of the HCM heart

Early characteristics of HCM include LV diastolic dysfunction, reduced LV distensibility, and hypercontractility. HCM patients who exhibit perfusion defects exhibited a longer regional time-to-peak wall-thickness-thinning rate, a measure of early diastolic dysfunction.^88^ Patients with ischaemia during exercise have a higher LV end diastolic pressure (LVEDP) and pulmonary artery wedge pressure, suggesting ischaemia relates to reduced LV distensibility.^79,80^ The eventual fall of coronary BF during rapid atrial pacing is associated with a substantial rise LVEDP.^73^

With disease progression, hypertrophy develops. In a study comparing HCM patients with mild and severe hypertrophy and controls, resting myocardial BF was the highest in the mild hypertrophy group, whereas the myocardial BF of the severe hypertrophy group and healthy controls was similar.^46^ This may initially result from ischaemia-mediated vasodilation in mild hypertrophy; however, as hypertrophy progresses, structural myocardial and vascular changes reduce BF, rendering compensatory mechanisms insufficient.

Hyperaemic myocardial BF correlated negatively with structural and functional parameters such as wall thickness, systolic thickening, LV end systolic diameter, LV mass, and LVEDP, and positively with functional parameters such as LV ejection fraction (LVEF) and fractional shortening (FS),^35,36,38,40,49–51,53,56^ although two studies did not find a relationship between hyperaemic myocardial BF and wall thickness, and LVOT obstruction^36^ and between perfusion defects and wall thickness.^88^ CFR was lower in patients with vasodilator-induced LV cavity dilation and patients who exhibited ischaemia also demonstrated reversible LV cavity dilation, suggesting an interplay between ischaemia, LV function and CFR.^45,80^ HCM patients with irreversible perfusion defects exhibit a larger LV cavity, an increased left atrial (LA) diameter, lower FS and lower maximal exercise oxygen consumption compared to patients without irreversible defects.^89^

Perfusion deficits and reduced BF are more common in areas with hypertrophy and become more prominent with increasing wall thickness, particularly during hyperaemia.^14,16,17,19,20,22–24,26,27,60,77^ Consequently, overall resting coronary BF correlates positively with LVM, and this association was the opposite for CFR.^60^ When comparing segments with and without hypertrophy (both without LGE), hypertrophic segments exhibited a lower myocardial perfusion. Overall, these studies imply a relationship between perfusion deficits and the degree of cardiac hypertrophy irrespective of fibrosis.^17^ Of note, two studies did not find a relationship between perfusion defects and hypertrophy.^30,32^

Regional differences in myocardial BF have been observed in HCM. Septal myocardial BF in the septum was lower than in the lateral wall,^50,54,55^ though in one study this difference disappeared during hyperaemia.^40^ Perfusion defects are most commonly found in the ventricular septum, but can also occur in other regions.^87,98^ In apical HCM, hyperaemic myocardial BF was significantly lower in the apical region, particularly in the endocardium, compared to septal and inferolateral regions.^44^ Relative CFR (the ratio between CFR of the LAD and CFR of the LCx) in HCM and HOCM patients was <1 at rest and >1 during stress, whereas in controls both ratios remained at 1. This pattern suggests a higher baseline septal BF and impaired vasodilatory reserve of the septal vessels under stress.^59^

Layer-specific differences show more pronounced perfusion impairment in the endocardial than the epicardial layers.^38,40^ Subendocardial hyperaemic myocardial BF and MPR were significantly reduced, even in non-hypertrophied and non-fibrotic segments.^33^ While resting myocardial BF was lower in both layers in HCM patients compared to controls, the endo/epi myocardial BF ratio is similar at rest but significantly reduced during stress, indicating impaired endocardial vasodilatory capacity.^38,51^ These stress-induced reductions were observed in both the septum and lateral wall. The blunted hyperaemic response became more severe with increasing end-diastolic wall thickness, especially in the endocardium.^15^ Moreover, with decreasing hyperaemic myocardial BF, the difference between endocardial and epicardial perfusion increased.^40^ In one study, resting myocardial BF was higher in the endocardium than in the epicardium, but this reversed during stress.^19^ The endo-to-epi hyperaemic myocardial BF ratio also inversely correlated with structural disease markers such as LVOT gradient, LV mass, and LVEDP, suggesting its potential as a severity marker.^38^ Lastly, patients with subendocardial ischaemia had significantly larger LA diameters compared to those without and exhibited a significantly greater exercise-induced percentage increase in LV end-systolic volume, suggesting impaired contractile reserve in ischaemic regions^82,90^

Obstructive HCM patients exhibit more and more pronounced perfusion abnormalities than non-obstructive patients,^26,27^ and a significant correlation between perfusion and LVOT diameter (reflecting the severity of LVOT narrowing) has been shown,^27^ although another study did not confirm this finding.^75^ HOCM patients exhibited lower coronary resistance and higher coronary BF at baseline as well as during rapid atrial pacing when compared to HCM patients without LVOT obstruction. Moreover, obstructive HCM patients reached their maximal coronary BF during rapid atrial pacing earlier than non-obstructive HCM patients.^74^ Patients with severe microvascular dysfunction (defined as minimum MPRI <1.0) more often exhibited LVOT obstruction,^19^ although this has not been reproduced elsewhere.^22^ Relative CFR (ratio between CFR of the LAD and CFR of the LCx) correlated inversely with LVOT gradient.^59^

#### 3.2.6 Link between vascular abnormalities and fibrosis

Myocardial fibrosis was observed more frequently in segments with a lower perfusion.^15,17,19,20,23–25,30,33^ Moreover, patients with LGE display a lower perfusion^14,18,22,29,32,34,35,43,95^ and perfusion defects were more common in patients who exhibit a higher LGE percentage (>15%).^99^ The area of hypoperfused myocardium was often larger compared to the LGE area.^17^ Moreover, segments without fibrosis, but adjacent to segments with fibrosis, showed a reduced hyperaemic myocardial BF when compared to remote segments, which contributes to the theory that P/LP gene variant-related reduced perfusion in non-hypertrophied myocardium precedes development of fibrosis.^17,35^ Even in non-fibrotic, non-hypertrophic segments a reduced perfusion was found.^23^ However, two studies did not find a relationship between perfusion and degree of fibrosis.^16,56^

#### 3.2.7 Link between vascular abnormalities and (ventricular) arrhythmias

Patients with HCM are at increased risk for ventricular arrhythmias, which may be related to perturbed ion homeostasis in cardiomyocytes, i.e. action potential remodelling, or structural remodelling of the heart, including hypertrophy and fibrosis. Myocardial fibrosis contributes to increased arrhythmia risk by creating areas of heterogeneous conduction that promote arrhythmogenic circuits. Perfusion defects can indirectly increase arrhythmia risk by ischaemia-mediated remodelling of the action potential, or cause fibrosis via cell death. Seven studies in our systematic review reported on the occurrence of ventricular arrhythmias. HCM patients with reduced myocardial BF or perfusion abnormalities were more likely to experience (non-sustained) ventricular tachycardia (NSVT) and QT dispersion, suggesting a potential prognostic role for perfusion as a marker of arrhythmic risk in HCM patients.^18,28,55,77,85^ In contrast, 2 studies did not report an association between perfusion and ventricular arrythmias.^52,54^ However, the stress heterogeneity (highest hyperaemic myocardial BF value divided by the lowest) was significantly higher in patients who developed ventricular arrhythmias.^54^

In addition to ventricular arrhythmias, atrial fibrillation (AF) is frequently observed in HCM patients, which may be related to impaired diastolic function and atrial remodelling. Two studies reported on atrial fibrillation and found that HCM patients with the lowest hyperaemic myocardial BF were more likely to have atrial fibrillation.^36,55^

#### 3.2.8 Link among vascular abnormalities, disease progression and mortality

HCM patients with LV systolic dysfunction or those who progressed to LV heart failure exhibited significantly greater impairments in cardiac perfusion compared to those without heart failure, and perfusion defects (irreversible, partially reversible or combined with patchy LGE) related to end-stage disease.^25,86,87^ HCM patients in the dilated (end-stage) phase of the disease showed a significantly lower perfusion than HCM patients in the non-dilated phase.^91^ In patients with a reduced ejection fraction, the perfusion defects mostly appeared in regions with a normal wall or slightly increased wall thickness, whereas in the patients with a normal ejection fraction, the defects were mostly in the hypertrophied regions,^87^ possibly showing that the ‘irreversible perfusion defect areas’ have a reduced thickness due to thinning and scarring of the wall. Hyperaemic myocardial BF positively correlated with rest systolic blood pressure, and HCM patients with a lower blood pressure (≤ 110 mmHg) exhibited significantly greater microvascular dysfunction.^56^ This association may be related to a reduced blood pressure as a consequence of advanced disease progression, or the presence of low blood pressure itself is causing reduced myocardial perfusion, leading to ischaemia.

A strong relationship between perfusion (hyperaemic myocardial BF and CFR) and unfavourable outcome has been established.^51,53,36^ These unfavourable outcomes include progression of NYHA class, LV systolic dysfunction, increase in LV dimensions, (life-threatening) ventricular arrhythmias, cardioembolic stroke, severe functional limitation, and death. Patients in the lowest tertile of hyperaemic myocardial BF showed a sevenfold increased risk of an unfavourable outcome compared to the middle and highest tertiles, suggesting that perfusion may be a potential marker for prognosis. A blunted hyperaemic myocardial BF in the lateral wall is associated with a higher mortality in HCM, possibly due to the fact that the disease of the LV is more extensive when it even reaches the lateral wall and therefore reflects a more progressed disease course.^55^ MB in HCM does not seem to increase the risk of sudden cardiac death or mortality due to other causes.^70^

#### 3.2.9 Link between vascular abnormalities and symptoms

Nine studies reported on the association between perfusion and symptoms. HCM patients who experienced symptoms exhibited more irreversible and partially reversible defects than asymptomatic HCM patients, while the overall amount of perfusion defects did not differ.^87^ Irreversible defects were associated with dyspnoea, fatigue, syncope, reduced exercise capacity, and severe complications, but not to survival, which could also be due to a small overall event rate.^89,96^ The prevalence of angina in patients with ischaemic scintigrams was higher than in non-ischaemic scintigrams (80% vs. 23%).^81^ Moreover, a more advanced NYHA class negatively correlated with resting and hyperaemic myocardial BF, and CFR^18,51,53^ and patients with the lowest hyperaemic myocardial BF were more likely to receive medical treatment.^36^ Of note, one study did not find a relationship between perfusion defects and symptoms.^96^

#### 3.2.10 Risk factors and co-morbidities

Diabetes mellitus (DM) and obesity have been shown to negatively impact the progression of HCM.^10,11,148^ Additionally, both conditions can contribute to or exacerbate (micro)vascular dysfunction.^149,150^ The number of studies that focused on risk factors and co-morbidities was limited, with one paper reporting on DM and one on cardiac energetics. HCM patients with DM exhibited a more diminished hyperaemic myocardial BF compared to HCM patients without DM, although it is important to note that the HCM-DM patient group also included patients with hypertension.^29^ Associations between myocardial perfusion and cardiac energetics (decreased PCr/ATP-ratios) were not established in HCM patients.^31^

#### 3.2.11 Effect of treatments on cardiac perfusion

When assessing myocardial BF in patients who received medical treatment vs. a myectomy, medically treated patients show a reduced rest and hyperaemic myocardial BF and CFR compared to surgically treated patients.^57^ In most HCM patients with perfusion defects, normalization of these defects occurs after myectomy.^78^ This suggests a positive effect of myectomy on myocardial perfusion, possibly due to decreased intracardial pressures. Percutaneous transluminal septal myocardial ablation (PTSMA) in HOCM patients without perfusion defects at baseline naturally exhibits small septal perfusion deficits shortly after PTSMA, but after mid-term follow-up, the perfusion increased, albeit not back to baseline.^97^ One study assessed the effect of short-term treatment (± 8 weeks) with Verapamil on perfusion, but no effect on myocardial BF or CFR was found,^58^ while another study assessed the effect of a 1-week treatment with Verapamil on perfusion defects, and showed improvement of the perfusion defects in 10 out of 15 patients.^98^ To assess the effect of disopyramide in HOCM patients, it was intravenously administered and caused a reduction in coronary BF and an increase in coronary resistance, while the CFR remained unchanged, indicating a coronary vasoconstrictive effect.^66^ Intracoronary administration of ACE inhibitors resulted in an increase in coronary BF and CFR in HOCM patients, whereas these effects did not occur during circulatory administration. This is possibly due to a diminished coronary artery perfusion pressure and an increase in LVOT gradient after circulatory ACE administration.^65^

#### 3.2.12 Peripheral vascular function

Although HCM is primarily a myocardial disease, vascular abnormalities are observed not only in the hypertrophied myocardium but also in non-hypertrophied regions and the peripheral vasculature, suggesting a generalized alteration in vascular function.^151^ Therefore, we also included studies assessing peripheral vascular function in HCM. A total of 12 studies were included and none performed genetic testing. Nine of the 12 studies compared HCM patients with healthy controls and all 9 found significant differences.

Three studies assessed the vascular response to orthostatic stress, of which two found vascular differences.^105,107,108^ The first study used tilt-induced volume unloading and found that HCM patients with vasovagal syncope showed significant hypotension and reduced cardiac output (CO). Compared to non-HCM patients with syncope, HCM patients had higher systemic vascular resistance (SVR), indicating an adequate vascular response but impaired CO.^105^ The other two used lower body negative pressure (LBNP): one study found paradoxical vasodilation in 40% of non-obstructive HCM patients, though no control group was included.^108^ The other study, which did include controls, showed that forearm vascular resistance (FVR) increased significantly less in HCM patients and paradoxically decreased in 28%, suggesting impaired vasoconstriction.^107^

Another study investigated the mechanism behind episodic syncope and found that hypotensive episodes in HCM were associated with a 36% drop in peripheral resistance, without changes in CO, heart rate, or stroke volume—indicating inappropriate vasodilation.^106^

Findings on resting vascular tone were inconsistent. One study reported increased baseline forearm BF,^72^ another found no difference,^103^ and a third reported reduced BF and increased FVR in HCM patients.^113^

Four studies examined vascular responses to pharmacological agents. One found reduced vasoconstrictive response to phenylephrine, a beta-adrenergic receptor agonist, in HCM patients, while responses to isoproterenol, acetylcholine, and nitroglycerin were unchanged.^103^ However, another study found no difference in response to phenylephrine,^107^ while the third reported reduced vasodilation to nitroglycerin, a non-endothelium-dependent vasodilator.^110^ The fourth study found an increased vasoconstrictive response to angiotensin II, which mediates smooth muscle cell contraction.^113^

Four studies assessed the vascular response to exercise. One showed that HCM patients had reduced CO and higher peripheral resistance, the latter potentially serving as a compensatory mechanism for the reduced CO.^104^ Another study comparing HCM patients with and without an abnormal blood pressure response found no significant difference in SVR, though small sample size limited conclusions as there seems to be a trend towards a lower vasoconstrictive response.^109^ The third study observed that controls exhibited improved systolic function during handgrip exercise (e.g. decreased end-systolic volume, increased EF and stroke volume), whereas HCM patients did not.^111^ In contrast, SVR increased in HCM but not in controls, again suggesting a compensatory mechanism. The fourth study found no difference in response to handgrip exercise between groups.^107^

Three studies evaluated flow-mediated dilation after occlusion. Two found no difference, but BF decreased significantly in HCM patients after 60 s of hyperaemia, indicating an inability to maintain vasodilation—possibly due to elevated baseline flow. The third study showed reduced maximal vasodilator capacity post-occlusion in HCM patients.^113^

Taken together, some studies support the theory of generalized vascular dysfunction in HCM, though findings are inconsistent and may partly reflect compensatory mechanisms rather than primary vascular abnormalities.

#### 3.2.13 Early vascular changes at asymptomatic disease stage

Two studies assessed vascular function in G+/Ph− individuals with CMR. One showing no significant differences in MPRI when compared to G− controls,^20^ while the other study found significantly lower stress myocardial BF in G+/Ph−, although rest myocardial BF and MPR did not differ significantly.^34^ Interestingly, in two studies assessing oxygenation, a reduced oxygenation response was observed in G+/Ph− individuals compared to controls, despite a preserved MPRI. This suggests that oxygenation deteriorates first.^20,21^

Young patients at risk for HCM (defined as the presence of HCM among a first-degree relative) (age range 18.9 ± 3.8) showed a similar rest and hyperaemic myocardial BF as controls, although it is important to note that only 43% of these individuals were genotype-positive.^16^

One study assessed myocardial BF with PET in variant carriers and found no significant differences in rest and hyperaemic myocardial BF, and CFR when compared to G− controls, although it is important to note that the control group was significantly older (53 vs. 35 years), which could have led to an underestimation of differences.^41^ Similar research is of interest in dilated cardiomyopathy (DCM), in which vascular changes have also been described.^152,153^ Compared with non-failing hearts, patients with DCM exhibit reduced capillary density, decreased transcript levels of VEGF, and impaired VE-cadherin/β-catenin expression.^152,153^ Notably, a substantial proportion of idiopathic DCM cases are attributable to genetic mutations, further supporting the concept that mutation-mediated mechanisms may contribute to endothelial dysfunction and vascular remodelling in genetic cardiomyopathies.

#### 3.2.14 Histological analyses

Histopathological analysis of patient-derived cardiac tissue samples is a common method to study microvascular changes associated with HCM, allowing for a detailed assessment of the microvasculature. The most prominent changes in cardiac microvasculature include decreased capillary density, thickening of the vessel wall, narrowing of the vessel lumen, and increased perivascular fibrosis. We included 29 studies that performed analyses of the coronary vasculature in HCM. The most common sample types included were autopsy hearts, hearts from end-stage patients that required transplantation (11 studies),^4,114–123^ and septal myectomy samples (11 studies).^124–134^ Other studies included different sample types such as endomyocardial biopsies (six studies),^130,135–138,140^ and blood (one study),^139^ and two studies compared end-stage heart tissue to septal myectomy tissue.^4,5^ Most studies compared their findings to control tissue samples obtained from non-failing donor hearts and endomyocardial biopsies from individuals with normal heart function. In eight studies, findings were compared with hearts of other cardiac diseases and conditions such as dilated cardiomyopathy, idiopathic cardiomyopathy, coronary telangiectasia, hearts of patients who died from sudden cardiac death, hypertensive hearts, and even late-stage HCM hearts showing features of dilated cardiomyopathy (DCM-like HCM).^4,116–119,135,136,138^ Consistently, more pronounced vessel wall thickening, narrowing of the vessel lumen, decreased capillary density, and myocardial bridges were found in HCM tissue samples compared to controls. When HCM samples were compared to a DCM heart, HCM tissue was found to have less severe microvascular changes.^116,136^ Capillary density was lower in end-stage heart failure samples compared to myectomy samples. Moreover, in myectomy samples, capillary density was significantly lower in females compared to males.^4^ Comparing an early vs. a late disease stage, one study found that fibrosis was more likely associated with microvascular alterations in end-stage failing heart samples compared to myectomy samples.^5^ Eleven studies assessed fibrosis in HCM samples, where microvascular dysfunction was positively correlated with the degree of fibrosis, indicating that decreased BF can lead to regional ischaemia and subsequently replacement fibrosis.^4,5,114,123–127,132,133,138^

Taken together, microvascular alterations and fibrosis were reported in most studies reviewed here. Microvascular alterations are confirmed in early and late disease stages, indicating that this is a process that already manifests in early disease stages, independent of fibrosis. Fibrosis, on the other hand, was mostly positively correlated with microvascular alterations in myectomy samples and end-stage heart samples.

Genetic testing was performed in only 6 out of 29 studies, resulting in 130 G+/Ph+ patients.^3,5,123,129,131,134,135^ The most commonly reported genes involved *MYBPC3* and *MYH7*. None of the studies performed comparisons between genotypes or G+ and G− tissue samples, and thus no conclusions can be drawn.

## 4. Discussion

Myocardial perfusion in HCM can be assessed using various imaging modalities, including PET-CT, CMR, angiography, scintigraphy, and SPECT. Among these, PET-CT, CMR, and angiography provide quantitative assessments, whereas scintigraphy and SPECT are typically limited to qualitative evaluations.

Perfusion abnormalities are common in HCM and are not only observed in regions of established hypertrophy and fibrosis but also in structurally unaffected areas. These abnormalities correlate with early disease markers, such as diastolic dysfunction and hypercontractility. At rest, BF in HCM patients is often similar to or higher than in healthy controls. However, during stress, the increase in myocardial perfusion is significantly blunted, resulting in a diminished myocardial perfusion reserve. This supports the hypothesis that HCM is characterized by impaired vasodilatory capacity. This limitation may reflect either a compensatory increase in resting flow due to early structural or metabolic changes (such as substrate shift, hypertrophy, myocyte disarray, and fibrosis), or a primary inability of the coronary vessels to dilate appropriately.

Interestingly, patients with mild hypertrophy tend to show increased myocardial BF compared to controls, while those with more advanced disease and severe hypertrophy demonstrate reduced perfusion. The most profound reductions are seen in areas with significant hypertrophy and fibrosis, particularly in the endocardial layers. These perfusion deficits have been linked to adverse clinical outcomes, highlighting their potential prognostic value. In addition to cardiac vascular abnormalities, peripheral vascular dysfunction has also been reported in HCM patients. However, the findings across these studies are inconsistent, and the underlying mechanisms remain incompletely understood.

Additionally, microvascular alterations including decreased capillary density, vessel wall thickening, and narrowing of the vessel lumen are consistently observed in HCM tissue across various disease stages and sample types, compared to controls. These findings suggest that microvascular dysfunction is an early and persistent feature of HCM pathology, potentially contributing to the development of myocardial fibrosis. The positive correlation between fibrosis and microvascular abnormalities further supports a pathogenic link, where ischaemia-induced replacement fibrosis perpetuates disease progression. In line with findings from other imaging modalities such as CMR (increased LGE is associated with reduced perfusion), perivascular fibrosis is associated with a higher degree of microvascular changes and abnormalities. Despite limited genetic data, the recurrent involvement of MYBPC3 and MYH7 emphasizes established genetic underpinnings, urging further research into genotype-phenotype correlations.

This review has limitations that should be acknowledged. First, because no meta-analysis was conducted, the results lack quantitative synthesis. Moreover, substantial methodological heterogeneity—including different modalities, small cohort sizes and the use of older datasets—combined with a limited number of longitudinal and interventional studies makes it difficult to draw firm conclusions. Second, all studies involving patients with hypertension were excluded to ensure a homogeneous cohort of individuals with HCM, resulting in the exclusion of a substantial number of potentially relevant studies. While this may have led to the exclusion of well-designed research, it was necessary to avoid confounding vascular findings related to hypertensive heart disease. However, not all studies explicitly reported the prevalence of hypertension in their cohorts. We chose to include studies that stated the exclusion of hypertensive patients in their methods, provided that no patients with hypertension were listed in the baseline characteristics. As a result, it remains possible that some studies may have inadvertently included individuals with hypertension.

Additionally, the proportion of studies that conducted genetic testing was low, raising concerns about the inclusion of patients with mixed aetiologies of left ventricular hypertrophy. This makes it more difficult to draw conclusions specifically about genetic hypertrophic cardiomyopathy. This limitation was particularly present in studies investigating peripheral vascular function, scintigraphy and SPECT, which further complicates the interpretation of vascular abnormalities as being specific to HCM. To further elucidate the role of vascular dysfunction in HCM, future studies should aim to assess both cardiac and peripheral vascular function in a large cohort across the full spectrum of disease—from genotype-positive/phenotype-negative individuals to those with advanced structural disease. Importantly, such studies should employ a combination of imaging modalities to allow for comprehensive evaluation and cross-validation of vascular measures. Genetic testing should be consistently performed to better correlate vascular findings with genotype and to identify early vascular alterations in asymptomatic carriers.

## Supplementary Material

cvag153_Supplementary_Data

## Data Availability

The data underlying this article will be shared on reasonable request to the corresponding author.
