# Supplementary material for: Vascular changes in hypertrophic cardiomyopathy
Source: Cardiovasc Res. 2026 Jul 15;122(13):1865–79. doi: 10.1093/cvr/cvag153 (PMC13604838; doi:10.1093/cvr/cvag153)
Supplement: Supplementary file 1 [file cvag153_supplementary_data.docx]

**SUPPLEMENTARY MATERIAL**

***Table S1****: Search terms.*

***Table S2:*** *Overview of reported patient characteristics, cardiac dimensions, and measures of myocardial perfusion of the included PET studies.*

***Table S3:*** *Overview of reported patient characteristics, cardiac dimensions, and measures of myocardial perfusion of the included CMR studies.*

## Table S1 – Search terms

## A) PubMed (4,436); (68)

| **Search** | **Query** | **Results** |
| --- | --- | --- |
| **#3** | **#**1 AND #2 | 4,436 |
| **#2** | **Search: "Cardiomyopathy, Hypertrophic"[Mesh] OR "Hypertrophic cardiomyopath*"[tiab] OR "HCM"[tiab] OR HOCM[tiab] OR "Asymmetric septal hypertroph*"[tiab] OR "Hypertrophic obstructive cardiomyopath*"[tiab] OR "Hypertrophic non-obstructive cardiomyopath*"[tiab] OR "Hypertrophic Cardiomyopath*"[tiab] OR "Hypertrophic Obstructive Cardiomyopath*"[tiab] OR "Hypertrophic non-obstructive cardiomyopathy*"[tiab] OR ( (mutat*[tiab] ) AND ("MYH7"[tiab] OR "Myosin binding protein C3"[tiab] OR "Troponin T"[tiab] OR "TnnT2"[tiab] OR "TnnI3"[tiab] OR "MYL2"[tiab] OR "Myosin heavy chain 7"[tiab] OR MYBPC3[tiab]) ) OR "HCM mutation carrier"[tiab] OR "Hypertrophic cardiomyopathy mutation carrier"[tiab] OR "Idiopathic hypertrophic subaortic stenosis"[tiab]** | [24,223](https://pubmed.ncbi.nlm.nih.gov/?term=%22Cardiomyopathy%2C+Hypertrophic%22%5BMesh%5D+OR%0A%22Hypertrophic+cardiomyopath%2A%22%5Btiab%5D+OR%0A%22HCM%22%5Btiab%5D+OR%0AHOCM%5Btiab%5D+OR%0A%0A%22Asymmetric+septal+hypertroph%2A%22%5Btiab%5D+OR%0A%22Hypertrophic+obstructive+cardiomyopath%2A%22%5Btiab%5D+OR%0A%22Hypertrophic+non-obstructive+cardiomyopath%2A%22%5Btiab%5D+OR%0A%0A%E2%80%9CHypertrophic+Cardiomyopath%2A%E2%80%9D%5Btiab%5D+OR%0A%E2%80%9CHypertrophic+Obstructive+Cardiomyopath%2A%E2%80%9D%5Btiab%5D+OR%0A%E2%80%9CHypertrophic+non-obstructive+cardiomyopathy%2A%E2%80%9D%5Btiab%5D+OR%0A%0A%0A%28%0A%28mutat%2A%5Btiab%5D+%29%0AAND%0A%28%E2%80%9CMYH7%E2%80%9D%5Btiab%5D++OR%0A%E2%80%9CMyosin+binding+protein+C3%E2%80%9D%5Btiab%5D+OR%0A%E2%80%9CTroponin+T%E2%80%9D%5Btiab%5D+OR%0A%E2%80%9CTnnT2%E2%80%9D%5Btiab%5D+OR+%0A%E2%80%9CTnnI3%E2%80%9D%5Btiab%5D+OR%0A%E2%80%9CMYL2%E2%80%9D%5Btiab%5D+OR%0A%E2%80%9CMyosin+heavy+chain+7%E2%80%9D%5Btiab%5D+OR%0AMYBPC3%5Btiab%5D%29%0A%29+OR%0A%E2%80%9CHCM+mutation+carrier%E2%80%9D%5Btiab%5D+OR%0A%E2%80%9CHypertrophic+cardiomyopathy+mutation+carrier%E2%80%9D%5Btiab%5D+OR%0A%0A%E2%80%9CIdiopathic+hypertrophic+subaortic+stenosis%E2%80%9D%5Btiab%5D&sort=relevance&ac=no) |
| **#1** | **Search: "blood supply" [Subheading] OR "Blood Vessels"[Mesh] OR "Vascular*"[tiab] OR "Circulatory system"[tiab] OR Capillar*[tiab] OR "Blood vessel*"[tiab] OR "blood supply"[tiab] OR "blood flow*"[tiab] OR "Endothelial function*"[tiab] OR "Endothelial dysfunction*"[tiab] OR "Arter*"[tiab] OR "vein*"[tiab] OR "Cardiovascular system"[tiab] OR Microvascula*[tiab] OR Vessel*[tiab] OR "Myocardial perfusion*"[tiab] OR "cardiac perfusion*"[tiab] OR "Perfusion imag*"[tiab] OR "Perfusion ind*"[tiab] OR "Angio*"[tiab]** | [2,712,379](https://pubmed.ncbi.nlm.nih.gov/?term=%22blood+supply%22+%5BSubheading%5D+OR%0A%22Blood+Vessels%22%5BMesh%5D+OR%0A%22Vascular%2A%22%5Btiab%5D+OR%0A%22Circulatory+system%22%5Btiab%5D+OR%0ACapillar%2A%5Btiab%5D+OR%0A%0A%22Blood+vessel%2A%22%5Btiab%5D+OR%0A%E2%80%9Cblood+supply%E2%80%9D%5Btiab%5D+OR+%0A%E2%80%9Cblood+flow%2A%E2%80%9D%5Btiab%5D+OR%0A%0A%E2%80%9CEndothelial+function%2A%E2%80%9D%5Btiab%5D+OR%0A%E2%80%9CEndothelial+dysfunction%2A%E2%80%9D%5Btiab%5D+OR%0A%0A%22Arter%2A%22%5Btiab%5D+OR%0A%E2%80%9Cvein%2A%E2%80%9D%5Btiab%5D+OR%0A%22Cardiovascular+system%22%5Btiab%5D+OR%0AMicrovascula%2A%5Btiab%5D+OR%0AVessel%2A%5Btiab%5D+OR%0A%0A%E2%80%9CMyocardial+perfusion%2A%E2%80%9D%5Btiab%5D+OR%0A%E2%80%9Ccardiac+perfusion%2A%E2%80%9D%5Btiab%5D+OR%0A%E2%80%9CPerfusion+imag%2A%E2%80%9D%5Btiab%5D+OR%0A%E2%80%9CPerfusion+ind%2A%E2%80%9D%5Btiab%5D+OR%0A%0A%E2%80%9CAngio%2A%E2%80%9D%5Btiab%5D&sort=relevance&ac=no) |

## B) Embase (8,226); (90)

| **Search** | **Query** | **Results** |
| --- | --- | --- |
| #3 | **#1 AND #2** | 8226 |
| #2 | **Search: 'hypertrophic cardiomyopathy'/exp OR ("Hypertrophic cardiomyopath*" OR "HCM" OR HOCM OR "Asymmetric septal hypertroph*" OR "Hypertrophic obstructive cardiomyopath*" OR "Hypertrophic non-obstructive cardiomyopath*" OR "Hypertrophic Cardiomyopath*" OR "Hypertrophic Obstructive Cardiomyopath*" OR "Hypertrophic non-obstructive cardiomyopathy*" OR ( (mutat* ) AND ("MYH7" OR "Myosin binding protein C3" OR "Troponin T" OR "TnnT2" OR "TnnI3" OR "MYL2" OR "Myosin heavy chain 7" OR MYBPC3) ) OR "HCM mutation carrier" OR "Hypertrophic cardiomyopathy mutation carrier" OR "Idiopathic hypertrophic subaortic stenosis"):ti,ab,kw** | 41,144 |
| #1 | **Search: 'vascularization'/exp OR 'blood vessel'/exp OR ("Vascular*" OR "Circulatory system" OR Capillar* OR "Blood vessel*" OR "blood supply" OR "blood flow*" OR "Endothelial function*" OR "Endothelial dysfunction*" OR "Arter*" OR "vein*" OR "Cardiovascular system" OR Microvascula* OR Vessel* OR "Myocardial perfusion*" OR "cardiac perfusion*" OR "Perfusion imag*" OR "Perfusion ind*" OR "Angio*"):ti,ab,kw** | 3,709,745 |

## C) WofS (4,564); (42)

| **Search** | **Query** | **Results** |
| --- | --- | --- |
| **#3** | **#1 AND #2** | 4,564 |
| **#2** | **TS=("Hypertrophic cardiomyopath*" OR "HCM" OR HOCM OR "Asymmetric septal hypertroph*" OR "Hypertrophic obstructive cardiomyopath*" OR "Hypertrophic non-obstructive cardiomyopath*" OR "Hypertrophic Cardiomyopath*" OR "Hypertrophic Obstructive Cardiomyopath*" OR "Hypertrophic non-obstructive cardiomyopathy*" OR ( (mutat* ) AND ("MYH7" OR "Myosin binding protein C3" OR "Troponin T" OR "TnnT2" OR "TnnI3" OR "MYL2" OR "Myosin heavy chain 7" OR MYBPC3) ) OR "HCM mutation carrier" OR "Hypertrophic cardiomyopathy mutation carrier" OR "Idiopathic hypertrophic subaortic stenosis")** | 29,823 |
| **#1** | **TS=("Vascular*" OR "Circulatory system" OR Capillar* OR "Blood vessel*" OR "blood supply" OR "blood flow*" OR "Endothelial function*" OR "Endothelial dysfunction*" OR "Arter*" OR "vein*" OR "Cardiovascular system" OR Microvascula* OR Vessel* OR "Myocardial perfusion*" OR "cardiac perfusion*" OR "Perfusion imag*" OR "Perfusion ind*" OR "Angio*")** | 2,883,734 |

**Table S2 – Baseline data, PET**

| **Study & Year** | **Country** | **Article DOI** | **Tracer** | **Which groups were included?** | **n** | **Genetic test performed?** | **If yes, % mutation positive?** | **If yes, which gene/mutation?** | **Average age** | **% male** | **Average BMI** | **Average BSA** | **MWT mm** | **LV mass (g)** | **LV mass (g)/**BSA | **LVEF %** | **% LVOT obstruction** | **LVOT gradient** | | **MBF rest** | **MBF stress** | **CFR/MPRI/MPR** | **Patients with LGE %** | **Average amount of LGE %** |
| --- | --- | --- | --- | --- | --- | --- | --- | --- | --- | --- | --- | --- | --- | --- | --- | --- | --- | --- | --- | --- | --- | --- | --- | --- |
| Perrone-Filardi, 1993 | US | 10.1016/0002-9149(93)90160-e. | H215O | Symptomatic HCM patients, similar septal and lateral thickening | 5 | No | - | - | 37 ± 17 | 60 | N.R. | N.R. | N.R. | N.R. | N.R. | 76 ± 11 | N.R. | - | | 0.90 ± 0.20 (septal and lateral) | N.R. | N.R. | N.R. | N.R. |
|  |  |  |  | Symptomatic HCM patients, more lateral than septal thickening | 5 |  |  |  | 35 ± 18 | 20 |  |  |  |  |  | 81 ± 6 |  | - | | 1.00 ± 0.30 (septal), 1.10 ± 0.40 (lateral) |  |  |  |  |
| Gistri, 1994 | IT | 10.1016/0002-9149(94)90404-9 | 13N-labeled ammonia | HCM patients treated with verapamil | 10 | No | - | - | N.R. | N.R. | N.R. | N.R. | 23 ± 6 | N.R. | N.R. | N.R. | N.R. | 15 ± 14 | | 0.92 ± 0.34 (pre-treatment) | 1.83 ± 0.89 (pre-treatment) | 2.00 ± 0.67 (pre-treatment) | N.R. | N.R. |
|  |  |  |  | HCM patients treated with placebo | 10 |  |  |  |  |  |  |  | 26 ± 5 |  |  |  |  | 19 ± 19 | | 0.83 ± 0.29 (pre-treatment) | 1.47 ± 0.61 (pre-treatment) | 1.79 ± 0.56 (pre-treatment) |  |  |
| Choudhury, 1997 | UK | 10.1093/oxfordjournals.eurheartj.a015090 | H215O | HCM patients | 12 | No | - | - | 34 (11) | 100 | N.R. | N.R. | 19 (5) | N.R. | N.R. | N.R. | N.R. |  | N.R. | 0.82 (0.23) | 1.64 (0.44) | 2.05 (0.61) | N.R. | N.R. |
|  |  |  |  | Secondary LVH | 16 |  |  |  | 64 (18) | 43.8 |  |  | 13 (1) |  |  |  |  |  |  | 1.17 (0.40) | 2.27 (0.60) | 2.06 (0.62) |  |  |
|  |  |  |  | Controls (matched to HCM group) | 15 |  |  |  | 36 (11) | 93.3 |  |  | N.R. |  |  |  |  |  |  | 0.94 (0.14) | 3.50 (0.95) | 3.81 (0.98) |  |  |
| Radvan, 1997 | UK | 10.1016/s0002-9149(97)00778-9 | H215O | HCM patients | 10 | No | - | - | 27 ± 5 | 100 | N.R. | N.R. | 19 ± 5 | N.R. | N.R. | N.R. | N.R. | - | | 0.80 ± 0.17 | 1.54 ± 0.45 | 1.96 ± 0.65 | N.R. | N.R. |
|  |  |  |  | Athletes  Controls | 10  10 |  |  |  | 24 ± 3  27 ± 5 | 100 100 |  |  | 13 ± 1 N.R. |  |  |  |  | - | | 0.74 ± 0.17 0.8 ± 0.2 | 2.69 ± 1.15 3.1 ± 0.8 | 3.58 ± 1.02 3.86 ± 0.65 |  |  |
| Lorenzoni, 1997 | IT and UK | 10.1093/oxfordjournals.eurheartj.a015204 | 13N-labeled ammonia and H215O | HCM patients with NSVT | 26 | No | - | - | N.R. | N.R. | N.R. | N.R. | 23 ± 5 | N.R. | N.R. | N.R. | N.R. | - | | 0.77 ± 0.29 | 1.34 ±0.62 | 1.78 ± 0.71 | N.R. | N.R. |
|  |  |  |  | HCM patients without NSVT | 58 |  |  |  |  |  |  |  | 21 ± 5 |  |  |  |  | - | | 0.88 ± 0.30 | 1.58 ± 0.61 | 1.83 ± 0.57 |  |  |
| Lorenzoni, 1998 | IT and UK | 10.1016/s0002-8703(98)70152-8 | 13N-labeled ammonia and H215O | HCM patients | 84 | No | - | - | 43 ± 12 | 73.8 | N.R. | N.R. | 22 ± 5 | N.R. | N.R. | N.R. | 10.7 | N.R. | | 0.84 ± 0.30 | 1.51 ± 0.62 | 1.81 ± 0.61 | N.R. | N.R. |
|  |  |  |  | Controls | 28 |  |  |  | 47 ± 9 | 53.6 |  |  | N.R. |  |  |  | 0 | - | | 1.00 ± 0.22 | 3.13 ± 1.12 | 3.23 ± 1.24 |  |  |
| Cecchi, 2003 | IT | 10.1056/NEJMoa025050 | 13N-labeled ammonia | HCM patients | 51 | No | - | - | 44 ± 13 | 71 | N.R. | N.R. | 22 ± 5 | N.R. | N.R. | N.R. | 16 | N.R. | | 0.84 ± 0.32 | 1.50 ± 0.69 | 1.84 ± 0.67 | N.R. | N.R. |
|  |  |  |  | Controls | 12 |  |  |  | 51 ± 8 | 33.3 |  |  | N.R. |  |  |  | 0 | **-** | | 1.00 ± 0.23 | 2.71 ± 0.94 | 2.7 ± 0.9 |  |  |
| Jörg-Ciopor, 2004 | SWI | 10.1016/j.jtcvs.2003.11.003 | 13N-labeled ammonia | HCM patients - myectomy | 11 | No | - | - | 56 ± 10 | N.R. | N.R. | N.R. | 19 ± 8 (post-op) | N.R. | N.R. | N.R. | N.R. | 8 ± 16 (post-op) | | 0.67 ± 0.15 | 1.55 ± 0.39 | 2.31 ± 0.40 | N.R. | N.R. |
|  |  |  |  | HCM patients - medically treated | 11 |  |  |  | 54 ± 13 |  |  |  | 24 ± 5 |  |  |  | 45.5 | 20 ± 32 | | 0.89 ± 0.21 | 1.49 ± 0.47 | 1.76 ± 0.58 |  |  |
|  |  |  |  | Controls | 15 |  |  |  | 34 ± 14 |  |  |  | N.R. |  |  |  | 0 | - | | 0.72 ± 0.20 | 3.04 ± 1.00 | 3.87 ± 0.92 |  |  |
| Knaapen, 2005 | NL | PMID: 15937301 | H215O | HCM patients | 21 | No | - | - | 56 ± 12 | 85.7 | N.R. | N.R. | 21 ± 6 | N.R. | N.R. | 65 ± 6 | N.R. |  | | 0.88 ± 0.23 | N.R. | N.R. | 81.0 | 14 ± 12 |
|  |  |  |  | Myocardial infarction patients | 12 |  |  |  | 58 ± 12 | 75 |  |  | 7 ± 2 |  |  | 30 ± 11 |  |  | | 0.92 ± 0.27 |  |  | 100 | 25 ± 16 |
|  |  |  |  | Controls | 6 |  |  |  | 50 ± 6 | 83.3 |  |  | 8 ± 1 |  |  | 58 ± 4 |  | - | | 0.94 ± 0.22 |  |  | 0 | 0 |
| Knaapen, 2006 | NL | 10.1016/j.nuclcard.2006.05.018 | H215O | HCM patients | 14 | No | - | - | 55 ± 12 | 92.9 | N.R. | N.R. | 22 ± 4 | N.R. | N.R. | 65 ± 5 | N.R. | - | | 0.78 ± 0.19 | N.R. | N.R. | N.R. | 16 |
| Olivotto, 2006 | IT | 10.1016/j.jacc.2005.10.050 | 13N-labeled ammonia | HCM patients | 51 | No | - | - | 44 ± 13 | 71 | N.R. | N.R. | 22 ± 5 | N.R. | N.R. | 69 ± 12 | 16 | N.R. | | 0.84 ± 0.31 | 1.50 ± 0.69 | N.R. | N.R. | N.R. |
|  |  |  |  | Controls | 12 |  |  |  | 51 ± 8 | 33.3 |  |  | N.R. |  |  | N.R. | 0 | - | | 1.00 ± 0.23 | 2.71 ± 0.94 |  |  |  |
| Tuunanen, 2007 | FI | 10.1016/j.nuclcard.2006.12.329 | H215O | HCM patients | 8 | Yes | 100 | 8x *TPM1* | 41 ± 14 | 37.5 | 25.5 ± 3.6 | N.R. | 14 ± 5.3 | 190 ± 80 | 102 ± 43 | 63 ± 9.7 | 0 | - | | 1.03 ± 0.29 | N.R. | N.R. | N.R. | N.R. |
|  |  |  |  | Controls | 36 | No | - | - | 46 ± 12 | 75 | 25.9 ± 3.2 |  | 8.7 ± 1.0 | 196 ± 38 | 101 ± 17 | 65 ± 5.3 | 0 | - | | 0.88 ± 0.19 |  |  |  |  |
| Soliman, 2007 | NL | 10.1136/hrt.2006.110460 | H215O | HCM obstructive patients | 14 | No | - | - | 53 ± 10 | 78.6 | N.R. | N.R. | 21 ± 4 | N.R. | 172 ± 18 | 64 ± 5 | 100 | 89 ± 31 | | 0.93 ± 0.17 | 2.42 **±** 0.57 | 2.67 ± 0.70 | N.R. | N.R. |
|  |  |  |  | Controls | 14 |  |  |  | N.R. | N.R. |  |  | N.R. |  | N.R. | N.R. | 0 | - | | 1.15 ± 0.14 | 4.56 ± 0.52 | 4.02 ± 0.71 |  |  |
| Knaapen, 2008 | NL | 10.1152/ajpheart.00233.2007 | H215O | HCM patients | 18 | No | - | - | 53 ± 11 | 72.2 | N.R. | N.R. | 18.9 ± 3.0 | N.R. | 102 ± 36 | 62 ± 7 | 66.7 | 49±34 | | 0.90 ± 0.21 | 2.26 ± 0.97 | 2.66 ± 1.32 | N.R. | N.R. |
|  |  |  |  | Controls | 10 |  |  |  | 49 ± 9 | 10 |  |  | N.R. |  | N.R. | N.R. | 0 | - | | 0.94 ± 0.21 | 2.93 ± 0.64 | 3.26 ± 1.05 |  |  |
| Sotgia, 2008 | IT | 10.2967/jnumed.107.050138 | 13N-labeled ammonia | HCM patients | 34 | No | - | - | 37 ± 17 | 67 | N.R. | N.R. | N. R. | 200 ± 94 | N.R. | 74 ± 12 | 9 | N.R. | | N.R. | 2.13 ± 1.03 | N.R. | 88 | 16.9 |
| Sciagrà, 2008 | IT | 10.1007/s12350-008-9005-5 | 13N-labeled ammonia | HCM patients with AF | 16 | No | - | - | 32 ± 11.6 | 72.7 | N.R. | N.R. | 24.1 ± 4.7 | N.R. | N.R. | 62 ± 19 | N.R. | 15.6 ± 19.4 | | N.R. | 1.23 ± 0.44 | N.R. | N.R. | N.R. |
|  |  |  |  | HCM patients without AF | 79 |  |  |  | 30.4 ± 14.4 | 88.9 |  |  | 12.1 ± 1.7 |  |  | 72 ± 10 |  | 13.4 ± 17.7 | |  | 1.87 ± 0.90 |  |  |  |
| Olivotto, 2011 | IT | 10.1016/j.jacc.2011.05.018 | 13N-labeled ammonia | Genotype positive HCM patients | 42 | Yes | 100 | 20 x *MYBPC*3, 11 x *MYH7*, 2 x *TNNT2*, 2 x *MYL2,* 1x *TPM1*, 6 x multiple | 39 ± 14 | 69 | N.R. | N.R. | 25 ± 5 | N.R. | N.R. | 70 ± 9 | N.R. | - | | N.R. | 1.7 (0.6-3.8) | N.R. | 96 | 16 ± 15 |
|  |  |  |  | Genotype negative HCM patients | 19 |  | 0 | - | 35 ± 15 | 79 |  |  | 26 ± 8 |  |  | 72 ± 8 |  | - | |  | 2.4 (1.1-5.6) |  | 67 | 17 ± 10 |
| Timmer, 2011 | NL | 10.1093/eurjhf/hfr135 | H215O | HCM mutation carriers | 15 | Yes | 100 | 15x *MYPBC3* | 35 ± 11 | 27 | 22.8 ± 2.1 | 1.82 ± 0.14 | 88 ± 15 | 93 ± 25 | N.R. | 52 ± 7 | 0 | - | | 1.19 +0.34 | 3.87 + 0.75 | 3.37 + 0.71 | N.R. | N.R. |
|  |  |  |  | Controls | 11 |  | 0 | - | 53 ± 3 | 64 | 24.6 ± 2.6 | 1.96 ± 0.10 | 95 ± 13 | 99 ± 21 | N.R. | 54 ± 10 | 0 | - | | 1.18 + 0.32 | 3.96 + 0.86 | 3.64 + 0.92 |  |  |
| Sciagrà, 2015 | IT | 10.1007/s00259-015-3101-y | 13N-labeled ammonia | HCM patients | 11 | No | - | - | 32 ± 11.6 | 72.7 | N.R. | N.R. | 24.1 ± 4.7 | N.R. | N.R. | 69 ± 7.9 | 0 | - | | 0.76 ± 0.20 | 1.77±0.52 | N.R. | N.R. | N.R. |
|  |  |  |  | Controls | 9 |  |  |  | 30.4 ± 14.4 | 88.9 |  |  | 12.1 ± 1.7 |  |  | 62 ± 8.8 | 0 | - | | 0.95 ± 0.27 | 2.91± 0.65 |  |  |  |
| Castagnoli, 2016 | IT | 10.1007/s00259-016-3465-7 | 13N-labeled ammonia | HCM patients | 100 | Yes | 65/100 | 36x *MYBPC3*, 19x *MYH7*, 10x other | 40 ± 16 | 71 | N.R. | N.R. | 23 ± 6 | N.R. | N.R. | 64 ± 13 | 12 | 13 ± 14 | | N.R. | 1.96 ± 0,86 | N.R. | N.R. | N.R. |
| Lu, 2020 | US | 10.1007/s12350-018-01521-x | 13N-labeled ammonia | HCM patients without LV cavity dilation | 53 | No | - | - | 51 ± 13 | 54 | 30 ± 8 | N.R. | 21 ± 5 | N.R. | N.R. | 66 ± 8 | 36 | 73 ± 51 | | 0.97 ± 0.32 | 2.15 ± 0.67 | 2.42 ± 0.78 | 66 | 13.8 ± 12.1 |
|  |  |  |  | HCM patients with LV cavity dilation | 55 |  |  |  | 44 ± 16 | 70 | 27 ± 3 |  | 22 ± 5 |  |  | 64 ± 7 | 22 | 43 ± 57 | | 0.98 ± 0.59 | 1.92 ± 0.67 | 2.33 ± 0.86 | 72 | 13.5 ± 8.1 |
| Lu, 2018 | US | 10.1016/j.amjcard.2018.01.022 | 13N-labeled ammonia | HCM patients without ventricular arrythmia | 110 | Yes | N.R. (8/38 patients in both groups together) | - | 52 ± 14 | 62 | 29 ± 6 | N.R. | 19 ± 5 | N.R. | 68 ± 24 | 69 ± 5 | 21 | 60 ± 48 | | 0.96 ± 0.47 | 2.09 ± 0.72 | 2.52 ± 0.80 | 60 | 14 ± 9 |
|  |  |  |  | HCM patients with ventricular arrythmia | 23 |  |  |  | 48 ± 15 | 55 | 29 ± 6 |  | 23 ± 6 |  | 87 ± 30 | 69 ± 5 | 40 | 71 ± 53 | | 0.96 ± 0.25 | 1.97 ± 0.52 | 2.20 ± 0.60 | 78 | 13 ± 11 |
| Calabretta, 2022 | IT | 10.1007/s12350-022-02911-y | 13N-labeled ammonia | Apical HCM patients | 12 | No | - | - | 44 (26-60) | 83.3 | N.R. | N.R. | 16.2 ± 4.5 | N.R. | N.R. | N.R. | 0 | - | | N.R. | N.R. | N.R. | N.R. | N.R. |
| Lu, 2023 | US | 10.1016/j.hroo.2023.07.009 | 13N-labeled ammonia and H215O | HCM patients ≤ 110mmHg | 80 | Yes | nov-23 | 5 x *MYBPC3*, 3 x *MYH7*, 1 x *MYL2*, 2 x *TNNI3/TNNT2* | 46 ± 15 | 55 | 29 ± 6 | N.R. | 21 ± 5 | N.R. | 73 ± 28 | 68 ± 10 | 32 | 58 ± 56 | | 0.81 ± 0.27 | 1.77 ± 0.53 | 2.28 ± 0.37 | 66 | 16 ± 11 |
|  |  |  |  | HCM patients 110-140 mmHg | 395 |  | 33/71 | 15 x *MYBPC3*, 8 x *MYH7*, 7 x *TNNI3/TNNT2*, 1 x *ACTN2/MYPN*, 1 x *MYL2*, 1 x *RYR2/CAV3* | 50 ± 14 | 68 | 29 ± 6 |  | 21 ± 6 |  | 77 ± 28 | 68 ± 9 | 31 | 70 ± 54 | | 0.92 ± 0.28 | 2.01 ± 0.61 | 2.33 ± 0.77 | 69 | 15 ± 13 |
|  |  |  |  | HCM patients > 140 mmHg | 215 |  | jun-27 | 2 x *MYPBC3*, 2 x *TNNI3/TNNT2*, 1 x *MYH7*, 1 x *RYR2/CAV3* | 60 ± 13 | 59 | 30 ± 7 |  | 21 ± 5 |  | 85 ± 32 | 70 ± 10 | 33 | 75 ± 55 | | 1.06 ± 0.49 | 2.28 ± 0.77 | 2.56 ± 0.94 | 68 | 13 ± 10 |

**Table S3 – Baseline data, CMR**

| **Study & Year** | **Country** | **Article DOI** | **Modality** | **Which groups were included?** | **n** | **Genetic test performed?** | **If yes, % mutation positive?** | **If yes, which gene/mutation?** | **Average age** | **% male** | **Average BMI** | **Average BSA** | **MWT mm** | **LV mass (g)** | **LV mass (g)/**BSA | **LVEF %** | **% with LVOT obstruction** | **LVOT gradient** | **Visual perfusion defects %** | **MBF rest** | **MBF stress** | **CFR/MPRI/MPR** | **% patients with LGE** | **Average amount of LGE (%)** |
| --- | --- | --- | --- | --- | --- | --- | --- | --- | --- | --- | --- | --- | --- | --- | --- | --- | --- | --- | --- | --- | --- | --- | --- | --- |
| Soler, 2006 | ES | 10.1097/00004728-200605000-00011 | Firstpass perfusion imaging | HCM patients | 53 | No | - | - | 49.55 ± 14.24 | 62 | N.R. | N.R. | 15.2± 4 | 207.5± 76.7 | N.R. | 74.5 ± 7.8 | 43.3 | N.R. | 30,2 | N.R. | N.R. | N.R. | 56.6 | N.R. |
| Petersen, 2007 | UK | 10.1161/CIRCULATIONAHA.106.657023 | Firstpass perfusion imaging | HCM patients | 35 | Yes | N.R. | N.R. | 44 ± 15 | 26 | N.R. | 1.96±0.24 | 16.7 ± 6.1 | N.R. | 88.1 ± 31.2 | 74 ± 9 | 14.3 | N.R. | N.R. | 0.71 ± 0.27 | 1.84 ± 0.89 | N.R. | N.R. | N.R. |
|  |  |  |  | Healthy controls | 14 | No |  |  | 39 ± 11 | 29 |  | 1.98±0.17 | 10.2 ± 1.4 |  | 64.9 ± 13.3 | 71 ± 4 | N.R. |  | N.R. | 0.85 ± 0.30 | 3.42 ± 1.76 |  |  |  |
| Melacini, 2007 | IT | 10.1016/j.ijcard.2007.06.023 | Firstpass perfusion imaging | HCM patients | 44 | Yes | 18 | 5x *MYH7*, 2x *MYBPC*3, 1x *TNNI3* | 47 ± 14 | 66 | N.R. | N.R. | 25 ± 6 | 94 ± 26 | N.R. | 56 ± 13 | 29 | N.R. | 46 | N.R. | N.R. | N.R. | 89 | N.R. |
| Barbosa, 2009 | BRA | 10.1590/s0066-782x2009001000017 | Firstpass perfusion imaging | HCM patients - nonobstructive | 10 | No | - | - | 32.2 | N.R. | N.R. | N.R. | N.R. | N.R. | N.R. | 71.0 ± 12.2 | 0 | N.R. | 30 | N.R. | N.R. | N.R. | N.R. | N.R. |
|  |  |  |  | HCM patients - obstructive | 11 |  | - | - | 36.5 | N.R. |  |  |  |  | N.R. | 77.6 ± 7.8 | 100 |  | 55 |  |  |  |  |  |
| Aquaro, 2011 | IT | 10.1016/j.cardfail.2011.01.006 | Coronary sinus flow | Controls | 20 | No | - | - | 48 ± 10 | 70 | N.R. | N.R. | 8 ± 3 | 129 ± 30 | 74 ± 25 | 64 ± 5 | 0 | - | N.R. | 0.92 ± 0.5 (measured by coronary sinus flow) | N.R. | N.R. | 0 | 0 |
|  |  |  |  | HCM patients | 62 |  | - | - | 47 ± 16 | 72 |  |  | 20 ± 5 | 197 ± 54 | 104 ± 28 | 69 ± 8 | 24 | N.R. |  | 0.56 ± 0.28 (measured by coronary sinus flow) |  |  | N.R. | 9.4 ± 13.5 |
|  |  |  |  | Acromegaly patients | 15 |  | - | - | 47 ± 12 | 60 |  |  | 13 ± 8 | 167 ± 60 | 93 ± 30 | 66 ± 7 | 0 | - |  | 1.36 ± 0.5 (measured by coronary sinus flow) |  |  | 0 | 0 |
| Hueper, 2012 | US | 10.1371/journal.pone.0041974 | Firstpass perfusion imaging | HCM patients | 70 | No | - | - | 51.7 [47.7. 55.8] | 80 | N.R. | N.R. | N.R. | N.R. | 98.5 [91.3, 105.7] | 80.1 [78.6, 81.6] | N.R. | 27.2 [19.8, 34,5] | N.R. | 1.54 [1.43, 1.65] (MBF corrected for the rate-pressure product) | N.R. | N.R. | 77 | 0 |
| Huang, 2013 | CN | 10.1016/j.crad.2013.01.003 | Firstpass perfusion imaging | HCM patients | 22 | No | - | - | 52.4 ± 12.5 | 66.7 | N.R. | 1.85 ± 0.13 | N.R. | N.R. | 80.4 ± 51.1 | 73.0 ± 7.0 | N.R. | N.R. | N.R. | N.R. | N.R. | N.R. | N.R. | N.R. |
|  |  |  |  | Healthy volunteers | 13 |  | - | - | 46.6 ± 15.1 | 61.5 |  | 1.76 ± 0.16 |  |  | 35.7 ± 8.0 | 64.5± 5.3 |  |  |  |  |  |  | 0 | 0 |
| Karamitsos, 2013 | UK | 10.1016/j.jacc.2012.12.024 | BOLD imaging | Normal controls | 20 | No | - | - | 39.6 ± 9.8 | 65 | N.R. | 1.93 ± 0.19 | 10.1 ± 1. | N.R. | 64.7 ± 9.9 | 61.9 ± 4.1 | 0 | - | N.R. | N.R. | N.R. | 1.8 ± 0.1 | 0 | 0 |
|  |  |  |  | Athletes | 11 | No | - | - | 31.5 ± 9.3 | 73 |  | 1.88 ± 0.17 | 12.4 ± 0.7 |  | 92.8 ± 16.1 | 64.9 ± 6.1 | 0 | - |  |  |  | 2.0 ± 0.1 | 0 | 0 |
|  |  |  |  | HCM mutation carriers | 10 | Yes | 100 | N.R. | 36.2 ± 14.2 | 40 |  | 1.71 ± 0.21 | 9.6 ± 0.9 |  | 60.4 ± 10.0 | 72.9 ± 7.8 | 0 | - |  |  |  | 1.7 ± 0.1 | 0 | 0 |
|  |  |  |  | HCM patients | 27 | Yes | N.R. |  | 48.2 ± 9.2 | 70 |  | 2.04 ± 0.22 | 19.3 ± 5.4 |  | 95.5 ± 40.2 | 73.6 ± 5.3 | 0 | - |  |  |  | 1.3 ± 0.1 | 67 | N.R. |
| Xu, 2014 | CN | 10.1016/j.ejrad.2014.01.008 | Firstpass perfusion imaging | Normal controls | 14 | No | - | - | 33.28 ± 18.44 | 50 | N.R. | N.R. | 6.23 ± 1.31 | 72.90 ± 24.61 | N.R. | 61.19 ± 2.89 | 0 | 0 | 0 | N.R. | N.R. | N.R. | N.R. | N.R. |
|  |  |  |  | NOHCM | 17 |  | - | - | 45.35 ± 14.38 | 58.8 |  |  | 13.87 ± 2.51 | 154.75 ± 44.67 | N.R. | 58.56 ± 8.3 | 0 | 21.66 ± 10.40 | 0 |  |  |  |  |  |
|  |  |  |  | HOCM | 25 |  | - | - | 44.92 ± 17.59 | 28 |  |  | 15.67 ± 2.65 | 165.22 ± 44.89 | N.R. | 63.46 ± 6.86 | 100 | 78.58 ± 27.31 | 12 |  |  |  |  |  |
| Ismail, 2014 | UK | 10.1186/s12968-014-0049-1 | Firstpass perfusion imaging | HCM - non-severe microvascular dysfunction | 24 | No | - | - | 58.9 (52.3. 67.5) IQR | 75 | N.R. | N.R. | 18.9 ± 4.5 | N.R. | 93.6 ± 24.9 | 72.0 ± 8.3 | 4.2 | N.R. | N.R. | N.R. | N.R. | N.R. | N.R. | N.R. |
|  |  |  |  | HCM - severe microvascular dysfunction | 11 |  | - | - | 52.0 (42.5. 63.6) IQR | 63.6 |  |  | 22.2 ± 4.0 |  | 103.4 ± 30.0 | 73.8 ± 6.5 | 27.3 |  |  |  |  |  |  |  |
| Gyllenhammar, 2014 | SE | 10.1093/ehjci/jeu137 | Coronary sinus flow | Controls | 12 | No | - | - | 22.8 ± 4.5 | 75 | N.R. | 1.9 ± 0.1 | 8.9 ± 0.5 | N.R. | 56 ± 3 | 55 ± 2 | 0 | - | 0 | N.R. | N.R. | N.R. | 0 | 0 |
|  |  |  |  | HCM risk | 14 | Yes | 43 | 4x *MYBPC3*, 1x *TNNT2*, 1x *MYH7* | 18.9 ± 3.8 | 50 |  | 1.8 ± 0.05 | 8.5 ± 0.3 |  | 46 ± 2 | 57 ± 1 | 0 | - | 0 |  |  |  | 0 | 0 |
|  |  |  |  | HCM | 10 | Yes | 70 | N.R. | 22.3 ± 6.4 | 80 |  | 2.0 ± 0.1 | 19.0 ± 2.4 |  | 75 ± 12 | 59 ± 3 | 0 | - | 67 (stress) |  |  |  | 70 | 5 ± 1 |
| Zhang, 2015 | CN | 10.1016/j.ejrad.2015.04.028 | First pass perfusion and Myocardial Transit Time | Controls | 18 | No | - | - | 36 (15–49) | 61.1 | 23 (21–25) | N.R. | 8 ± 1 | 155.3 ± 46.9 | N.R. | 69.2 ± 12.8 | N.R. | N.R. | N.R. | N.R. | N.R. | N.R. | 0 | 0 |
|  |  |  |  | HCM patients with LGE | 9 |  | - | - | 37 (20–51) | 58.3 | 24 (22–27) |  | 12 ± 6 | 197.8 ± 68.1 | N.R. | 68.5 ± 9.8 |  |  |  |  |  |  | 0 | 0 |
|  |  |  |  | HCM patients without LGE | 15 |  | - | - | N.R. | N.R. | N.R. |  | 15 ± 4 | 251.2 ± 78.7 | N.R. | 71.3 ± 14.6 |  |  |  |  |  |  | 100 | N.R. |
| Jablonowski, 2015 | SE | 10.1007/s00246-015-1214-5 | Firstpass perfusion imaging | Controls | 9 | No | - | - | 21 (16–30) | 78 | N.R. | N.R. | 10 (6–11) | N.R. | 59 (37–74) | 57 (42–63) | 0 | - | 0 | N.R. | N.R. | N.R. | 0 | 0 |
|  |  |  |  | HCM risk | 15 | Yes | 33 | 4x *MYBPC3*, 1x *TNNT2* | 18 (14–26) | 53 |  |  | 9 (7–11) |  | 46 (37–56) | 57 (52–66) | 0 | - | 0 |  |  |  | 0 | 0 |
|  |  |  |  | HCM | 12 | Yes | 58 | 3x *MYBPC3*, 3x *MYH7*, 1x *TCAP* | 20 (12–30) | 83 |  |  | 17 (14–36) |  | 65 (43–158) | 62 (36–77) | 0 | - | 73 (stress) |  |  |  | 73 | 2.9 (0.5–12) - median |
| Dass, 2015 | UK | 10.1093/eurheartj/ehv120 | Firstpass perfusion imaging | HCM patients | 35 | Yes | 74 | 18x *MYBPC3*, 8x *MYH7* | 44 ± 12 | 50 | N.R. | N.R. | 17.5 ± 5.8 | N.R. | 90+40 | 75 ± 0.47 | 0 | 0 | N.R. | N.R. | N.R. | 1.2+0.41 (midventricular septum) | N.R. | N.R. |
|  |  |  |  | Healthy volunteers | 20 | No | - | - | 40 ± 9 | 55 |  |  | 7.6 ± 1.5 |  | 66+9 | 63 ± 4 | N.R. | N.R. |  |  |  | 1.8+0.56 (midventricular septum) | 0 |  |
| Yin, 2017 | CN | 10.1007/s10554-017-1189-9 | Firstpass perfusion imaging | HCM patients | 47 | No | - | - | 49.7 ± 13.3 | 77 | N.R. | N.R. | N.R. | 143.1 ± 55.5 | N.R. | 61.2 ± 11.5 | N.R. | N.R. | N.R. | N.R. | N.R. | N.R. | 61.7 | N.R. |
|  |  |  |  | Healthy controls | 21 |  | - | - | 44.2 ± 14.8 | 62 |  |  | 5.42±1.61 | 93.5 ± 25.2 | N.R. | 59.9 ± 6.7 |  |  |  |  |  |  | 0 | 0 |
| Tezuka, 2018 | JPN | 10.1007/s00380-017-1088-y | Firstpass perfusion imaging | HCM patients | 37 | No | - | - | 60.5 ± 10.9 | 62.2 | N.R. | N.R. | 18.1 ± 4.1 | N.R. | 134.5 ± 64.5 | 66.1 ± 9.3 | 13.5 | N.R. | N.R. | N.R. | N.R. | 1.5 ± 0.5 | 91.9 | N.R. |
|  |  |  |  | LVH patients (caused by HT or AOS) | 24 |  | - | - | 64.8 ± 10.8 | 75 |  |  | 15.4 ± 2.0 |  | 103.1 ± 36.6 | 65.3 ± 8.8 | N.R. |  |  |  |  | 2.2 ± 0.9 | 20.8 |  |
|  |  |  |  | Normal subjects | 20 |  | - | - | 56.8 ± 17.8 | 60 |  |  | N.R. |  | 79.4 ± 26.8 | 59.4 ± 4.6 | - | - |  |  |  | 2.4 ± 0.9 | 0 |  |
| Grover, 2019 | AUS | 10.1093/ehjci/jey220 | BOLD imaging | MYBPC3 positive HCM patients | 20 | Yes | 100 | 20x *MYBPC3* | 45 ± 16 | 90 | N.R. | 2.5 ± 0.2 | 21 ± 5 | N.R. | 95 ± 31 | 78 ± 6 | 10 | N.R. | N.R. | N.R. | N.R. | N.R. | 100 | N.R. |
|  |  |  |  | MYBPC mutation positive siblings (LVH-) | 18 | Yes | 100 | 18x *MYBPC3* | 38 ± 14 | 44 |  | 2.2 ± 0.2 | 8 ± 1 |  | 48 ± 12 | 68 ± 10 | 0 | - |  |  |  |  | 0 | 0 |
|  |  |  |  | MYBPC3 mutation negative siblings | 11 | Yes | 0 | - | 39 ± 15 | 9 |  | 1.9 ± 0.2 | 7 ± 1 |  | 42 ± 1 | 64 ± 7 | 0 | - |  |  |  |  | 0 | 0 |
|  |  |  |  | Normal controls | 11 | No | - | - | 42 ± 9 | 54 |  | 1.8 ± 0.1 | 7 ± 1 |  | 65 ± 12 | 69 ± 5 | 0 | - |  |  |  |  | 0 | 0 |
| Komuro, 2021 | JPN | 10.1536/ihj.20-479 | Firstpass perfusion imaging | HCM- VT group | 15 | No | - | - | 63 ± 14 | 67 | 24 ± 5.2 | N.R. | 20 ± 6 | N.R. | N.R. | 66 ± 3 | N.R. | N.R. | N.R. | N.R. | N.R. | N.R. | 100 | N.R. |
|  |  |  |  | HCM - non-VT group | 16 |  | - | - | 61 ± 19 | 31 | 25 ± 3.7 |  | 18 ± 2 |  | N.R. | 63 ± 3 |  |  |  |  |  |  | 94 |  |
| Jex, 2022 | UK | 10.2337/dc22-0083 | Firstpass perfusion imaging | Healthy volunteers | 15 | No | - | - | 60 ± 12 | 67 | 25 ± 3 | N.R. | 10 ± 1 | 99 ± 27 | 54 ± 11 | 63 ± 4 | 0 | - | N.R. | 0.68 ± 0.03 | 2.06 ± 0.42 | 3.19 ± 0.79 | 0 | 0 |
|  |  |  |  | HCM | 18 | Yes | 33 | 6x *MYH7*, 2x *MYBPC3* | 59 ± 10 | 78 | 29 ± 5 |  | 20 ± 2 | 173 ± 63 | 90 ± 27 | 70 ± 9 | 1 | N.R. |  | 0.59 ± 0.19 | 1.74 ± 0.44 | 3.09 ± 1.06 | 100 | 4 ± 4 |
|  |  |  |  | HCM-DM | 20 | Yes | 30 | 2x *MYH7*, 1x *MYBPC3*, 1x *ACTC1*, 1x *TNNI3* | 64 ± 9 | 65 | 32 ± 6 |  | 21 ± 4 | 187 ± 73 | 92 ± 40 | 67 ± 9 | 1 |  |  | 0.69 ± 0.16 | 1.39 ± 0.42 | 2.04 ± 0.82 | 100 | 10 ± 8 |
| Das, 2022 | UK | 10.1093/ehjci/jeab210 | Firstpass perfusion imaging | HCM patients | 20 | Yes | 25 | 3x *MYH7*, 1x *TNNI3*, 1x *MYBPC3* | 48 ± 18 | 40 | N.R. | 1.9 ± 0.2 | 19 ± 5 | N.R. | 81 ± 41 | 67 ± 6 | 30 | N.R. | N.R. | N.R. | 1.4 ± 0.5 | 2.1 ± 0.8 | N.R. | 5.9 ± 10.1 |
|  |  |  |  | Controls | 10 | No | - | - | 27 ± 9 | 40 |  | 2.2 ± 0.4 | 11 ± 1 |  | 61 ± 16 | 64 ± 4 | - | - |  | 0.6 ± 0.1 | 2.5 ± 0.6 | 4.0 ± 0.9 | 0 | 0 |
| Joy, 2023 | UK | 10.1161/CIRCULATIONAHA.123.063835 | Firstpass perfusion imaging and diffusion tensor imaging | Healthy volunteers | 28 | No | - | - | 34 (32–39) | 46 | 24 (22–26) | 1.8 ± 0.3 | 9.3 (8.2–10.2) | N.R. | 53 (45–68) | 66 (63–68) | - | - | 0 | 0.77(0.68-0.86) | 2.77 ± 0.62 | 3.47(2.90-3.75) | 0 | 0 |
|  |  |  |  | Subclinical HCM | 77 | Yes | 100 | 46x *MYBPC3*, 18x *MYH7*, 5x *TNNI3*, 5x *TNNT2*, 1x *MYL2*, 1x *TPM1*, 1x *CSRP3* | 31 (23–40) | 40 | 25 (22–28) | 1.9 ± 0.2 | 9.6 (8.6–10.6) |  | 51 (43–60) | 71 (67–74) | - | - | 28 | 0.80(0.66-0.93) | 2.46 ± 0.54 | 3.03(2.52-3.75) | 8 | N.R. |
|  |  |  |  | G+LVH+ | 51 | Yes | 100 | 28x *MYBPC3*, 13x *MYH7*, 4x *TNNI3*, 4x *TNNT2*, 1x *CSRP3*, 1x *TNNC1* | 52 (37–59) | 69 | 25 (24–28) | 1.9 ± 0.2 | 17.2 (15.4–21.1) |  | 77 (67–92) | 79 (74–83) | N.R. | N.R. | 100 | 0.69(0.59-0.83) | 1.77 ± 0.52 | 2.59(1.96-3.19) | 92 |  |
|  |  |  |  | G-LVH+ | 50 | Yes | 0 | - | 59 (51–65) | 84 | 26 (24–28) | 2.0 ± 0.2 | 17.2 (15.9–21.7) |  | 96 (81–126) | 79 (75–82) |  |  | 82 | 0.64 (0.55–0.79) | 1.59 ± 0.48 | 2.30 (2.01–2.96) | 96 |  |

**Table S4 – Risk of bias assessment (ROBINS-E)**
Summary Risk of Bias Heatmap for HCM Pathology Studies by Study Design. Traffic light plot summarizing overall ROBINS-E risk.

**Table S5 – Summary of risk of bias (ROBINS-E).**
Stacked bar graph showing risk of bias judgments for each of the 7 ROBINS-E domains plus overall risk**.**
